# Development of new magnetic nanocomposite designed by reduced graphene oxide aerogel and HKUST-1, and its catalytic application in the synthesis of polyhydroquinoline and 1,8-dioxo-decahydroacridine derivatives

**DOI:** 10.1038/s41598-023-48674-5

**Published:** 2023-12-21

**Authors:** Naghmeh Farzaneh, Fateme Radinekiyan, Mohammad Reza Naimi-Jamal, Mohammad G. Dekamin

**Affiliations:** 1 Research Laboratory of Green Organic Synthesis and Polymers, Department of Chemistry, Iran University of Science and Technology, P.O. Box 16846-13114, Tehran, Iran; 2 Pharmaceutical and Heterocyclic Compounds Research Laboratory, Department of Chemistry, Iran University of Science and Technology, P.O. Box 16846-13114, Tehran, Iran

**Keywords:** Catalysis, Organic chemistry

## Abstract

In this study, new magnetic reduced graphene oxide aerogel/HKUST-1 nanocomposite was designed and synthesized given the transformation of graphene oxide sheets to three-dimensional reduced graphene oxide aerogel, the in-situ magnetization of aerogel substrate, and the in-situ formation of HKUST-1 particles. Apart from characterizing the chemistry and structure of the designed magnetic nanocomposite (FT-IR, EDX, ICP, FE-SEM, DLS, XRD, VSM, and TG analyses), its catalytic performance was evaluated in the one-pot synthesis of biologically active 1,8-dioxo-decahydroacridine and polyhydroquinoline derivatives. The combination of magnetized reduced graphene oxide aerogel and HKUST-1 in the form of a new heterogeneous magnetic nanocatalyst was accompanied by a high synergetic catalytic effect in the symmetric and unsymmetrical Hantzsch condensation reactions. Compared to previous research studies, the pharmaceutical 1,8-dioxo-decahydroacridine and polyhydroquinoline derivatives can be synthesized using a partial amount of this nanocatalyst with a high percentage of yields in a short reaction time.

## Introduction

**Figure 1:**
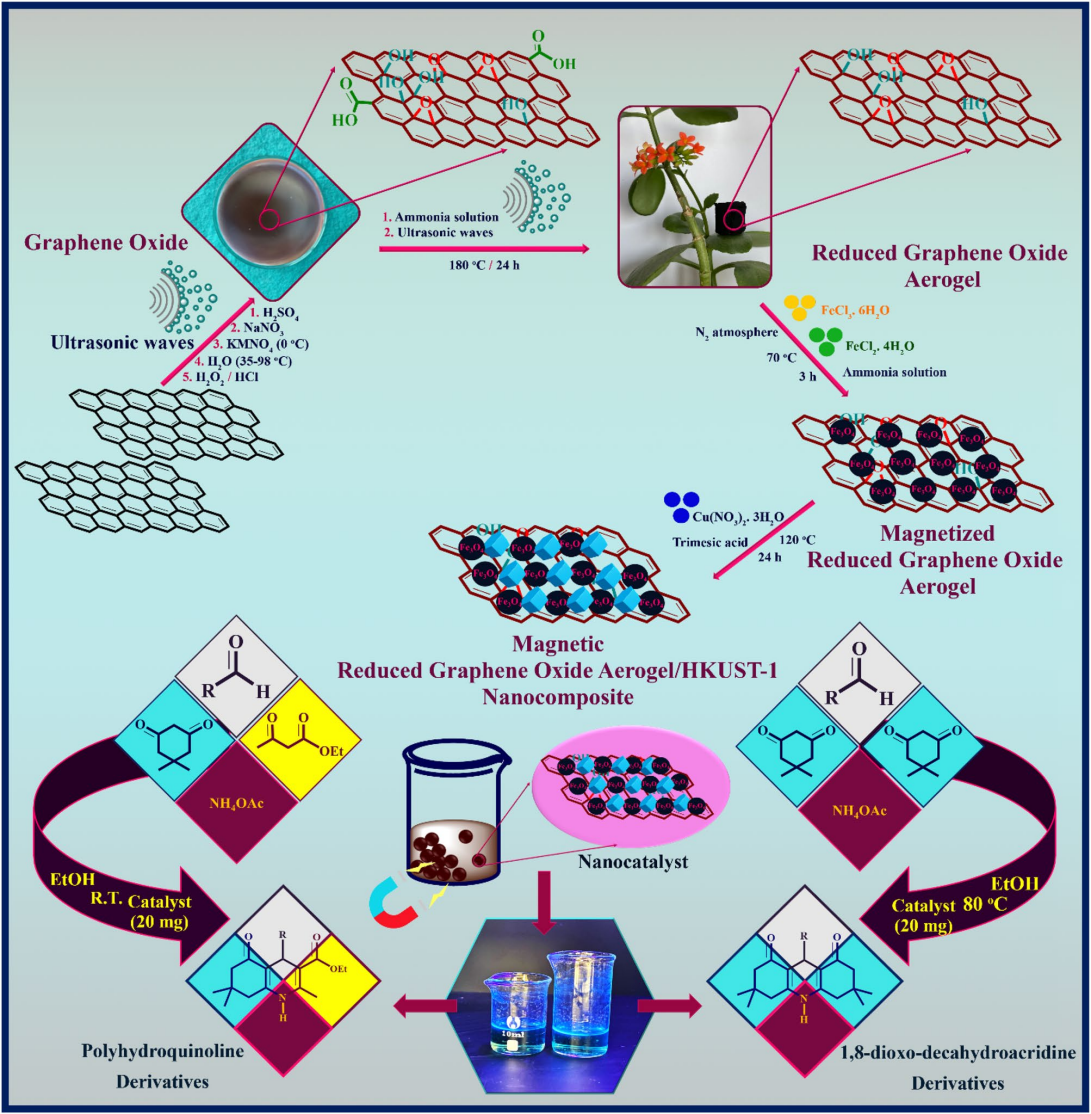
Synthesis process of magnetic rGO aerogel/HKUST-1 nanocomposite and its catalytic performance in the synthesis of Polyhydroquinoline and 1,8-dioxo-decahydroacridine derivatives.

Aerogels as air-filled, solid-sate, and lyophilized forms of hydrogels with three-dimensional assemble of microporous and mesoporous networks are designed and synthesized by various chemical precursors such as metals, metal oxides, carbides, amorphous carbon, and carbon allotropes^1,2^. These types of material with metastable characteristics can develop a diversity of new physical and chemical properties^3^. In terms of the type of building materials, bond strength, and the presence of dopants in the porous matrix, the application of these three-dimensional networks has been extended in catalysis science, filters and membranes, biomedical and chemical sensors, and adsorbents^2^. Among this diversity, graphene-based aerogels, the known lightest three-dimensional structures, have been a longstanding of interest due to their unique and exceptional features such as remarkable adsorption capacity, specific surface area, electrical conductivity, thermal resistance, and high mechanical strength^4,5^. In this context, one of the most appropriate building precursors to fabricate aerogel structures is graphene oxide (GO), the oxidized derivative of graphene sheets. Thanks to the oxygen-containing functional groups of GO sheets at both basal plans and edges, these functional sheets can react non-covalently and covalently with different compounds to generate new materials with specific efficiency and tailored applicability^6^. According to the colloidal stability of GO sheets, applying the hydrothermal process and reducing the oxygen-containing functional groups by different reducing agents (NH_3_, hydrazine, reducing sugar, ascorbic acid, Na_2_S, HI, NaHSO_3_, and transition metal chlorides)^2,7^, can cross-link the individual GO sheets and convert them into reduced graphene oxide (rGO) hydrogel^2^. Next, the formation of rGO aerogel is performed by freeze-drying and removing the trapped water molecules without collapsing the solid structure^2^. Particularly, this multifunctional structure can act as highly potent catalytic support to design and fabricate advanced and newly-discovered compositions^8,9^. Alongside graphene-based aerogels, metal-organic frameworks (MOFs) as porous coordination polymers are derived from the spontaneous self-assembly of metal ions and organic ligands. According to their distinctive features including intriguing structures with high activated surface area, high crystallinity, permanent porosity with adjustable pore size, tunable functionality, and designable topology, the application of these multifunctional structures has extended and developed in various fields such as gas sorption, sensors, separation, biomedicine, and etc^10^. In addition to this, these forefront and ideal platforms have been highlighted exclusively as new heterogeneous catalytic solids because of their unprecedented structural diversity, the presence of uncoordinated metal sites and available organic struts, the inherent hybrid organic-inorganic nature, and well-inferred porosity^11^. In this regard, one of the well-investigated coordination polymers in MOFs family is [Cu_3_(BTC)_2_] MOF (BTC = benzene-1,3,5-tricarboxylate), also termed HKUST-1 (MOF-199)^12^. This prototypical carboxylate MOF contains a large surface area, high pore volume, an extended cubic network, and excellent chemical stability. It can coordinate with water molecules using its unsaturated copper (II) sites^13^. On the other side, this MOF as a solid Lewis acid catalyst can be applied for the separation process, chromatography stationary phases, multicomponent reactions^14^, and as well, gas storage^15^. Over the past decade, in order to design and generate a diversity of chemical libraries containing multifarious molecular structures, multicomponent reactions have emerged as practical and systematic approaches with revolutionary changes in chemistry^16^. To synthesize heterocyclic compounds, these highly potent chemical reactions have been pioneers due to their high atomic economy, conducting the reaction in a shorter time, and compliance with green chemistry principles^17^. In this connection, considering the privileged importance of N-heterocyclic polyhydroquinoline and 1,8-dioxo-decahydroacridine compounds in the remedial and pharmaceutical functions, particularly, antitubercular, neuroprotective, antidiabetic, analgesic, and anticancer activities, the innovative chemical methods have been developed to synthesize these valuable compounds^18^. So far, a variety of synthesis protocols and different catalysts like L-proline^19^, CuBr^20^, salicylic acid^21^, and etc. have been proposed; but in this way, some drawbacks and difficulties such as low yield percentage of products, tedious work-up procedures, and designing catalysts with low efficiency have made the synthesis process of these biologically functional molecules challenging. As developed recently, the exclusive interest in Fe_3_O_4_ magnetic nanoparticles (Fe_3_O_4_ MNPs) have revealed an impressive role in biomedicine and catalysis science due to their convenient separation instead of centrifugation and filtration steps, providing an appropriate surface area, and as well, the formation of new heterogeneous magnetic-based composites^22^. According to these detailed descriptions, the research purpose of this study is to design and synthesize a new magnetic nanocomposite based on rGO aerogel, Fe_3_O_4_ MNPs, and HKUST-1 MOF structures. The synthesis process of magnetic rGO aerogel/HKUST-1 nanocomposite is followed by four main steps including the formation of GO sheets, reduction and conversion of GO sheets to rGO aerogel, in-situ magnetization of rGO aerogel substrate, and in-situ formation of HKUST-l MOF. According to specific and unique features of these qualified structures, it is hypothesized that the presence of rGO aerogel substrate, HKUST-1 MOF, and their combination in the structure of this new magnetic nanocomposite can be accompanied by an enhanced catalytic activity; therefore, the catalytic performance of magnetic rGO aerogel/HKUST-1 nanocomposite as a new heterogeneous nanocatalyst is evaluated in the one-pot condensation reaction of polyhydroquinoline (**5a-q**) and 1,8-dioxo-decahydroacridine (**6a-j**) derivatives (Fig. 1).

## Results and discussion

Following the preparation of magnetic rGO aerogel/HKUST-1 nanocomposite, characterizing its structural features including the formation of new functional groups, structural elements, morphology, magnetic properties, the crystalline phase, and thermogravimetric behaviour are evaluated using FT-IR, EDX, ICP, FE-SEM, DLS, VSM, XRD, TG analyses in each synthesis step. Besides, the catalytic performance of magnetic rGO aerogel/HKUST-1 nanocomposite as a new heterogeneous nanocatalyst is examined in the one-pot synthesis of biologically active polyhydroquinoline and 1,8-dioxo-decahydroacridine derivatives. The results are determined as follows.

### Characterization of magnetic rGO aerogel/HKUST-1 nanocomposite

#### FT-IR analysis

**Figure 2:**
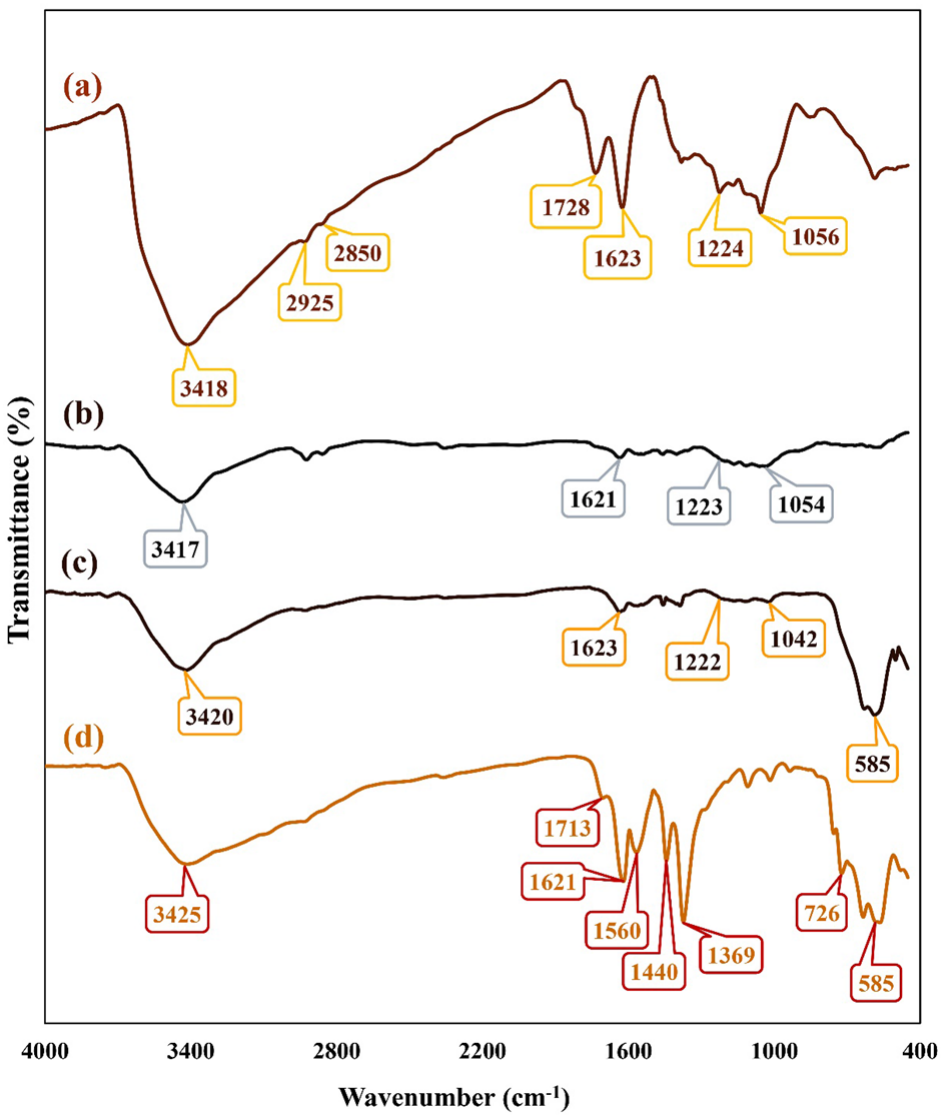
FT-IR spectra of (**a**) GO, (**b**) rGO aerogel, (**c**) magnetic rGO aerogel, and (**d**) magnetic rGO aerogel/HKUST-1 nanocomposite.

To characterize the formation of new functional groups and monitor the synthesis process in each step, FT-IR analysis was taken. Figure 2a shows FT-IR spectrum of synthesized GO substrate. As indicated, a broad band at the region of 3418 cm^−1^, a small absorption band at 1380 cm^−1^, and two small absorption bands at 2925 cm^−1^ and 2850 cm^−1^ are related to the stretching and bending vibration modes of hydroxyl groups^23,24^ and as well, asymmetric and symmetric CH_2_ stretching vibration modes^23^. The formation of carboxylic acid groups and the presence of graphitic domains are confirmed by observing two sharp absorption bands^23,25^, C=O stretching vibration mode at 1728 cm^−1^and C=C stretching vibration mode at 1623 cm^−1^. Moreover, observing two absorption bands at 1056 cm^−1^ and 1224 cm^−1^ is attributed to the C-O stretching vibration mode of epoxy and alkoxy groups^25^ and also, C-OH stretching vibration mode of alcohol groups formation^23^. As indicated in the FT-IR spectrum of rGO aerogel (Fig. 2b), given the reduction process and transformation of GO sheets to rGO aerogel, a dramatic decrease can be observed in the intensity of oxygen-containing functional group's absorption bands (C=O, O-H, C-O)^26^. In the third synthesis step, by magnetizing rGO aerogel and the in-situ formation of Fe_3_O_4_ MNPs, a sharp absorption band at 585 cm^−1^ is corresponded to the Fe–O stretching vibration mode and confirms the presence of synthesized Fe_3_O_4_ MNPs (Fig. 2c)^27^; and besides, in comparison to second synthesis step, it can be mentioned that the intensity of broad absorption band related to O-H stretching vibration mode (3420 cm^−1^) have increased^28^. Alongside of these descriptions, the formation of magnetic rGO aerogel/HKUST-1 nanocomposite was accompanied by the appearance of new absorption bands. As can be seen in Fig. 2d, the presence of an absorption band at 726 cm^−1^ related to the Cu-O stretching vibration mode, confirms the metal linker coordination^29^. Furthermore, the assigned absorption bands at 1369 cm^−1^, 1440 cm^−1^, 1560 cm^−1^, and 1621 cm^−1^ correspond to the symmetric and asymmetric stretching vibration modes of trimesic acid structure^30^. The assigned absorption band at 1621 cm^−1^ has covered the C=C stretching vibration mode of rGO aerogel substrate. In addition to this, a small absorption band at 1705 cm^−1^ confirms the presence of benzene-1,3,5-tricarboxylate structure^30^.

#### EDX and ICP analyses

**Figure 3:**
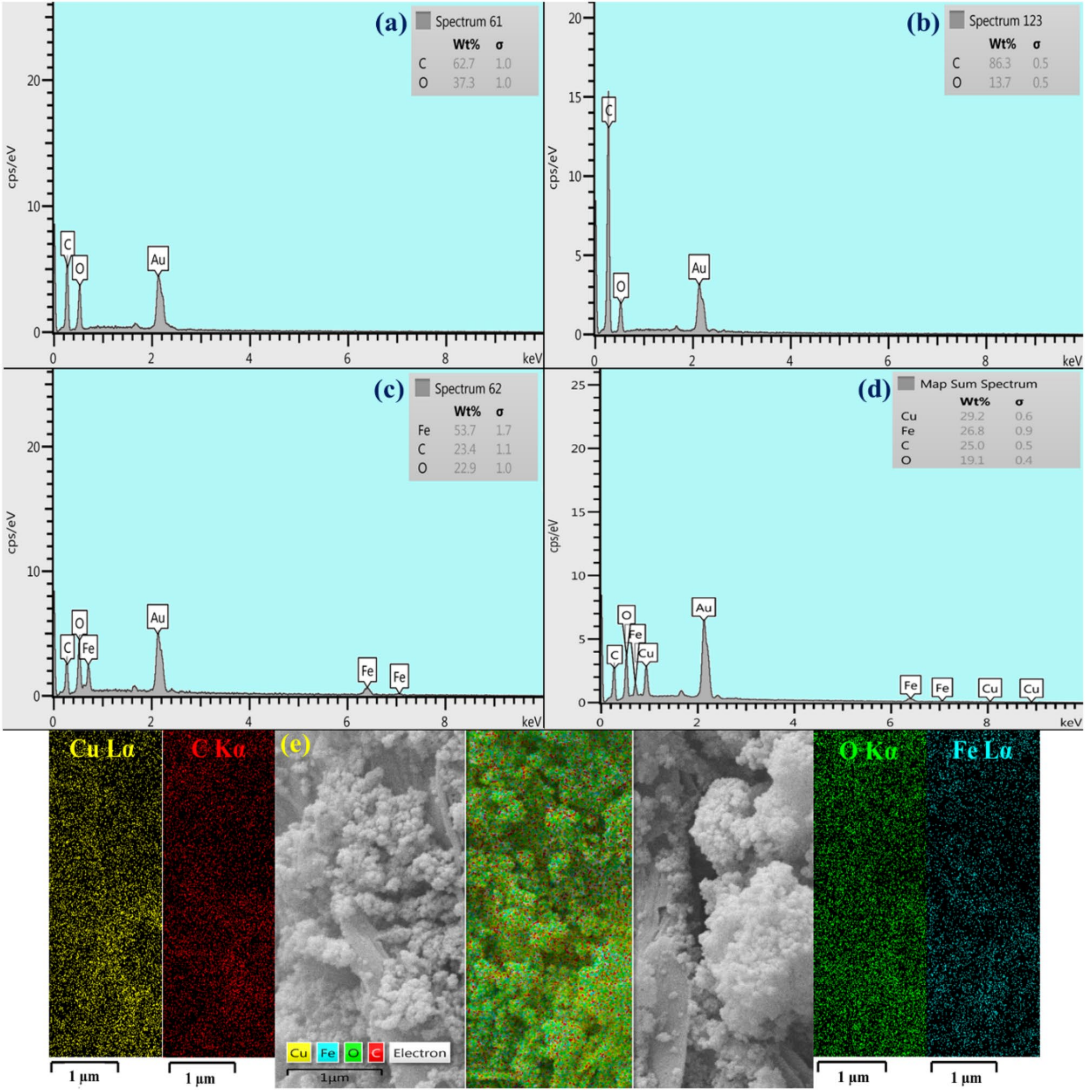
EDX spectra of (**a**) GO, (**b**) rGO aerogel, (**c**) magnetic rGO aerogel, (**d**) magnetic rGO aerogel/HKUST-1 nanocomposite, and (**e**) elemental mapping images of magnetic rGO aerogel/HKUST-1 nanocomposite.

The qualitative study of elemental composition and weight percentage of each synthesis step was performed by EDX analysis (Fig. 3a–e). As can be observed in Fig. 3a, the presence of two oxygen and carbon peaks can be attributed to the GO structure. Figure 3b shows the EDX spectrum of rGO aerogel. Although the EDX analysis is considered as a qualitative spectral technique, however, in comparison to Fig. 3a, the weight percentage of structural elements (carbon, oxygen) has changed; therefore, it can be deduced that the reduction of oxygen-containing functional groups and transformation of GO to rGO aerogel have well conducted. Following the in-situ magnetization process of rGO aerogel substrate, the presence of two iron peaks can be related to the synthesized Fe_3_O_4_ MNPs (Fig. 3c). Apart from mentioned structural elements, given the formation of magnetic rGO aerogel/HKUST-1 nanocomposite, in Fig. 3d, the two copper peaks can confirm the presence of HKUST-1. The amount of copper in the final composite was determined by ICP method as 17.2%, which is well comparable to the EDX data. In addition to this, the distribution pattern of observed structural elements has determined by elemental mapping images (Fig. 3e).

#### FE-SEM imaging

**Figure 4:**
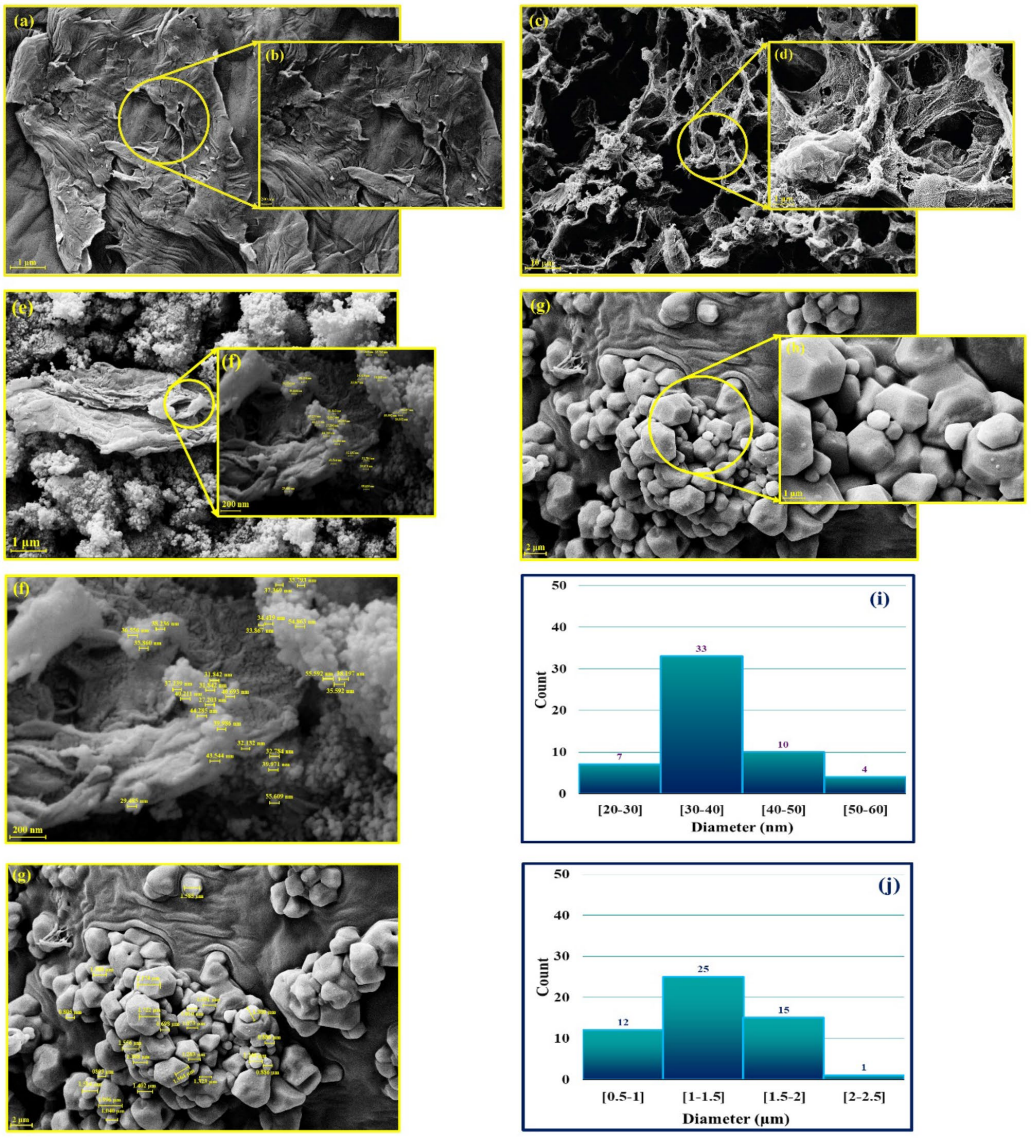
FE-SEM images of (**a**-**b**) GO, (**c**-**d**) rGO aerogel, (**e**–**f**) magnetic rGO aerogel, (**g**-**h**) magnetic rGO aerogel/HKUST-1 nanocomposite, (**i**) size distribution histogram chart of synthesized Fe_3_O_4_ MNPs in the structure of rGO aerogel substrate using Digimizer image analysis software, and (**j**) size distribution histogram chart of HKUST-1 particles in the structure of magnetic rGO aerogel/HKUST-1 nanocomposite using Digimizer image analysis software.

According to the fabrication process of magnetic rGO aerogel/HKUST-1 nanocomposite, the FE-SEM imaging was taken to monitor the morphology and structure from each synthesis step (Fig. 4a–j). As illustrated in Fig. 4a,b, the layer-by-layer GO structure can be well observed; and also, in some regions, the GO sheets are aligned parallel to each other. After the reduction process and freeze-drying step, the layer-by-layer assembly of GO sheets has changed to the channel-like structure with a considerable porosity (Fig. 4c,d). This hierarchically porous structure can provide a large specific surface area for the in-situ growth of nanoparticles^31,32^. In the next synthesis step, given the in-situ magnetization of prepared rGO aerogel substrate, the Fe_3_O_4_ MNPs with almost sphere shape have covered the pores of rGO aerogel (Fig. 4e,f). In addition to this, the average size of Fe_3_O_4_ MNPs is estimated between 30 nm to 40 nm due to the size distribution histogram chart (Fig. 4f,i). By the in-situ preparation of the HKUST-1 particles using hydrothermal method and formation of designed magnetic nanocomposite, the grown HKUST-1 particles with hexagonal shapes and an estimated average size of 1 µm to 1.5 µm are well characterized according to the size distribution histogram chart (Figs. 4g,h,j)^33^.

#### DLS analysis

Following the FE-SEM imaging, DLS analysis was performed from the bare Fe_3_O_4_ MNPs and HKUST-1 particles to evaluate and compare the results obtained from FE-SEM imaging. As indicated in the supplementary information file, Fig. S1a,b, in comparison to magnetized rGO aerogel with an estimated average size of Fe_3_O_4_ MNPs (30 nm–40 nm), most of the bare Fe_3_O_4_ MNPs with an average size of 74 nm (Fig. S1a) were characterized by DLS analysis. This difference is associated with the presence of rGO aerogel substrate in the synthesis process of Fe_3_O_4_ MNPs. The size of magnetic nanoparticles can be controlled by the presence of graphene derivatives, and as well, it can be mentioned that the presence of a graphene-based matrix can prevent the uncontrolled aggregation of magnetic nanoparticles due to its high specific surface area^34,35^. After the formation of magnetic rGO aerogel/HKUST-1 nanocomposite and estimating the average size of HKUST-1 particles (1 µm–1.5 µm) by FE-SEM imaging and using Digimizer image analysis software, the bare HKUST-1 particles with 1.47 µm average size (Fig. S1b) were observed by DLS analysis. This approximate reduction in the size of HKUST-1 particles is attributed to the presence of magnetized rGO aerogel. The graphene-based structures can strongly control the morphology of MOF particles and reduce their size. Controlling the size of MOF particles can tune the structural features of final composites and improve their practical performance^36^.

#### VSM analysis

**Figure 5:**
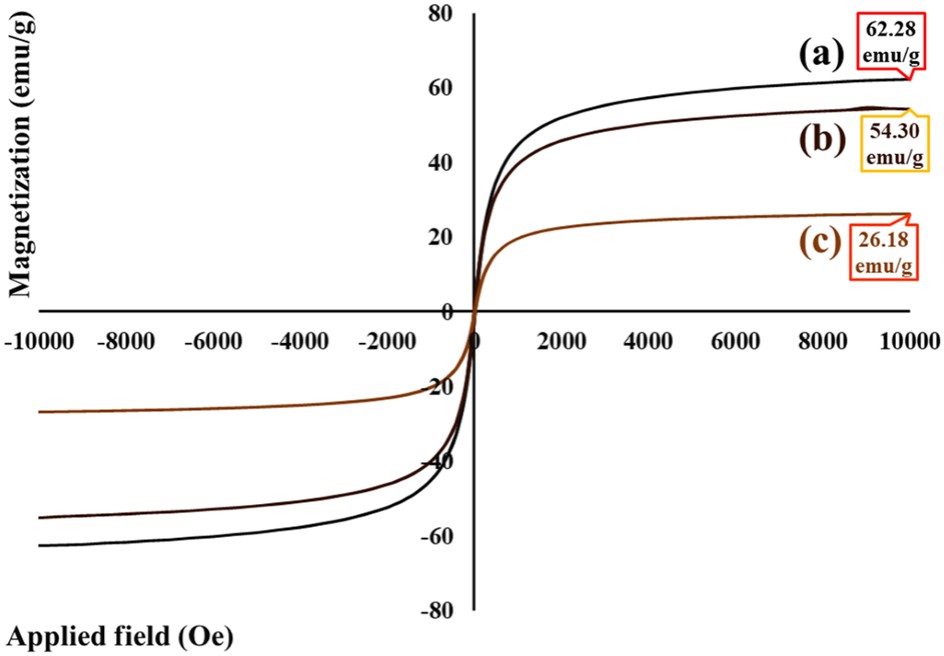
Hysteresis loop curves of (**a**) bare Fe_3_O_4_ MNPs, (**b**) magnetized rGO aerogel, and (**c**) magnetic rGO aerogel/HKUST-1 nanocomposite.

As a general rule, distinctive parameters including iron-group crystalline structure, core size, shell thickness, interparticle distance, and interparticle and interparticle interactions can verify the magnetic properties of nanoparticles^37^. As could be seen in Fig. 5a–c, the saturation magnetization value (M_s_) of bare Fe_3_O_4_ MNPs, magnetized rGO aerogel, and magnetic rGO aerogel/HKUST-1 nanocomposite is determined by their hysteresis loop curves. In comparison to the saturation magnetization value of bare Fe_3_O_4_ MNPs (62.28 emu/g) (Fig. 5a), this value for magnetized rGO aerogel and magnetic rGO aerogel/HKUST-1 nanocomposite has decreased to 54.30 emu/g (Fig. 5b), and 26.18 emu/g (Fig. 5c); which can be related to the presence of rGO aerogel substrate in the in-situ magnetization process, the in-situ formation of HKUST-1 particles, and their partial covering by the magnetized rGO aerogel structure respectively.

#### XRD pattern

**Figure 6:**
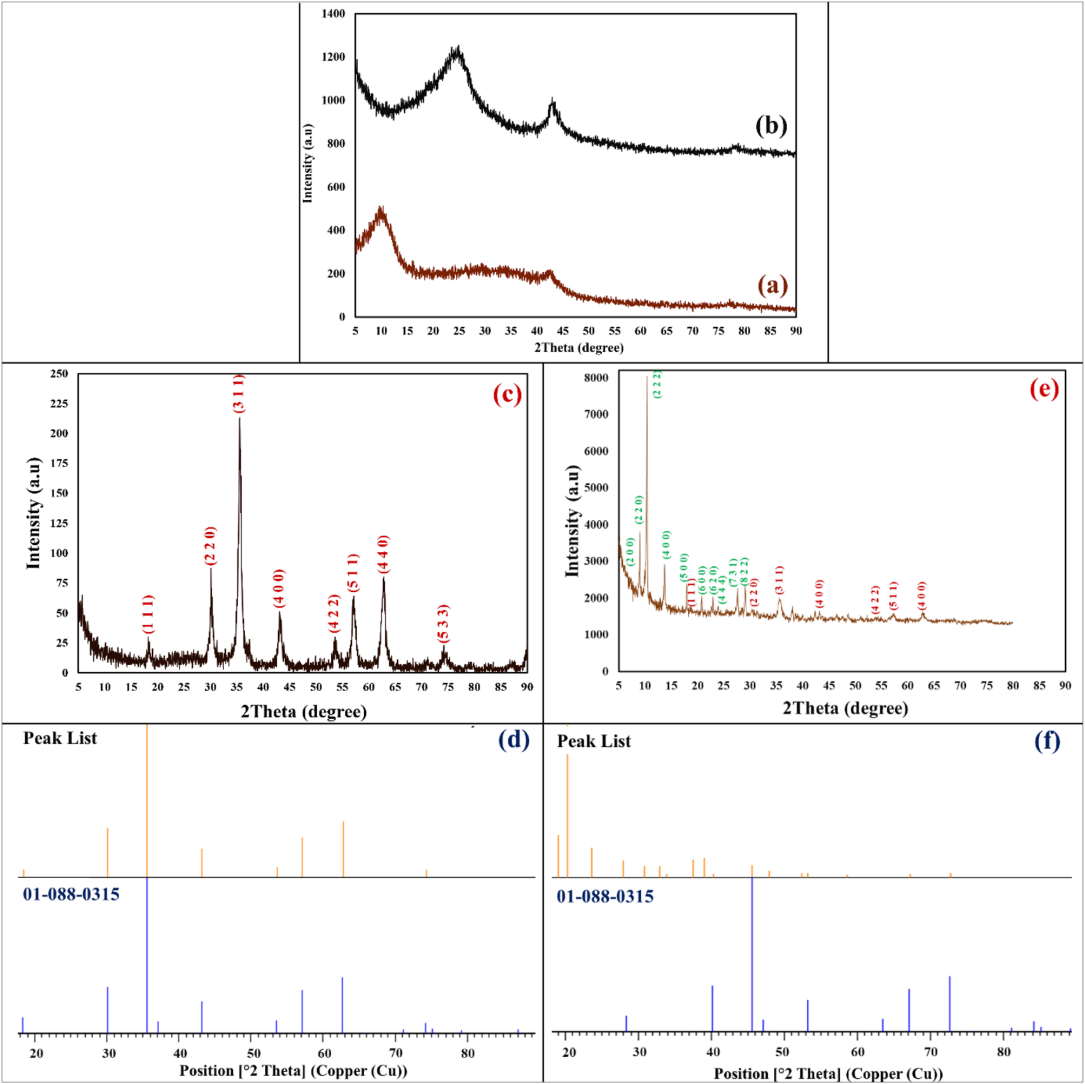
XRD patterns of (**a**) GO, (**b**) rGO aerogel, (**c**) magnetic rGO aerogel, (**d**) reference of synthesized Fe_3_O_4_ MNPs in the structure of magnetized rGO aerogel, (**e**) XRD pattern of magnetic rGO aerogel/HKUST-1 nanocomposite, and (**f**) reference of synthesized Fe_3_O_4_ MNPs in the structure magnetic rGO aerogel/HKUST-1 nanocomposite.

The crystalline phase and structure from each synthesized step of designed magnetic rGO aerogel/HKUST-1 nanocomposite was studied (Figs. 6a–f). As illustrated in Fig. 6a,b, the two peaks identified at determined diffraction angles (2Ө = 9.9°, 42.6°) confirm the complete oxidation of graphite to GO, its crystallographic plane (2Ө = 9.9°), and plane of graphene (2Ө = 42.6°)^38^. In the second synthesis step, a broad peak characterized at 2Ө = 24.6°, corresponds to the GO reduction process and the transportation of GO structure to rGO aerogel^39^. By the in-situ magnetization of rGO aerogel, the observed crystalline peaks at 2Ө = 18.41°, 30.15°, 35.58°, 43.21°, 53.62°, 57.11°, 62.79°, and 74.37° are indexed perfectly to the standard pattern of Fe_3_O_4_ MNPs (JCPDS card No. 01–088-0315)^40^. As well as, these identified peaks can be assigned with their Miller indices (1 1 1), (2 2 0), (3 1 1), (4 0 0), (4 2 2), (5 1 1), (4 4 0), and (5 3 3) (Fig. 6c,d). After the in-situ preparation of HKUST-1 particles, and the formation of magnetic rGO aerogel/HKUST-1 nanocomposite, apart from characterizing the standard pattern of Fe_3_O_4_ MNPs in the structure of magnetic nanocomposite, the identified peaks at 2Ө value including 6.44°, 9.01°, 10.35°, 13.69°, 17.93°, 20.78°, 22.91°, 23.88°,27.58°, 28.87, and as well, their Miller indices including (2 0 0), (2 2 0), (2 2 2), (4 0 0), (5 0 0), (6 0 0), (6 2 0), (4 4 4), (7 3 1), and (8 2 2) can be attributed to the formation of HKUST-1 particles (Fig. 6e,f)^41^.

#### Thermogravimetric analysis

**Figure 7:**
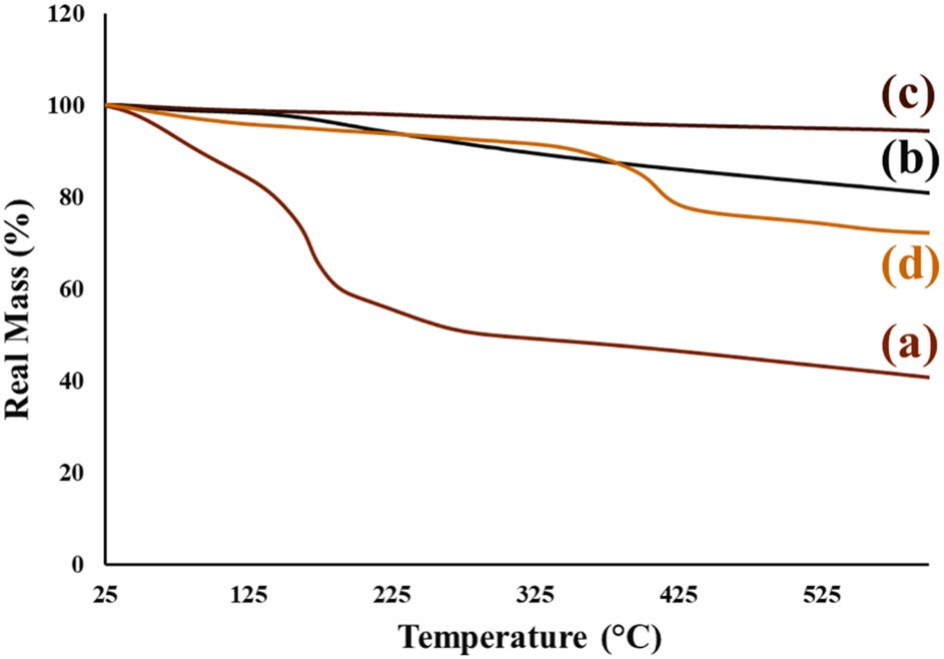
TG curves of (**a**) GO, (**b**) rGO aerogel, (**c**) magnetic rGO aerogel, and (**d**) magnetic rGO aerogel/HKUST-1 nanocomposite.

The thermal behaviour and thermogravimetric stability were studied from each synthesis step (Fig. 7a–d). Figure 7a shows the TG curve of GO sheets. As can be seen, the first mass reduction (almost 15%) from 25 ^o^C to almost 121 °C can be related to the moisture present and the evaporation of water molecules trapped inside the layer-by-layer GO structure^42^. In the following, the mass reduction dramatically continues up to approximately 300 °C (almost 35%); which can be attributed to the decomposition and pyrolysis of known oxygen-containing functional groups (epoxy, hydroxyl, and carboxylate groups)^43^. As illustrated in Fig. 7b, compared to the thermogravimetric curve of GO, the mass reduction and weight alternations are not noticeable; therefore, it can be deduced that the GO sheets reduction and their transformation to rGO aerogel have conducted and most of the oxygen-containing functional groups attached, are reduced during the hydrothermal process, yielding a better thermal stability^44^. Apart from the reduction process, by magnetizing the rGO aerogel structure, no specific mass reduction can be assigned at temperature range of 25 °C-600 °C. Therefore, it can be concluded that the emergence of Fe_3_O_4_ MNPs can increase the thermal stability of rGO aerogel substrate (Fig. 7c). After the formation of magnetic rGO aerogel/HKUST-1 nanocomposite, and the growth of HKUST-1 particles, in Fig. 7d, the first mass reduction (almost 5%) at temperature range of 25 °C to approximately 120 °C can be attributed to the evaporation of water molecules adsorbed in pores of HKUST-1 particles^45^. The second low mass reduction at temperature range of 125 °C to around 300 °C can be ascribed to the decomposition of low-quality HKUST-1 crystals^45^. The third and dramatic mass reduction (almost 20%), which starts at 315 °C, can be related to the complete decomposition of HKUST-1 particles and remaining of some resulting components such as Cu_2_O, and CuO^45^.

### Catalytic application of magnetic rGO aerogel/HKUST-1 nanocomposite

#### Optimization of reaction parameters in the one-pot synthesis of polyhydroquinoline derivatives

To evaluate the catalytic activity of magnetic rGO aerogel/HKUST-1 nanocomposite, a one-pot four-component condensation reaction of polyhydroquinoline derivatives was attended and tested. A model reaction mixture containing benzaldehyde (0.4 mmol), ethyl acetoacetate (0.5 mmol), dimedone (0.5 mmol), and ammonium acetate (2 mmol) was applied to optimize the different reaction parameters such as the amount of catalyst, temperature, and solvent (See the supplementary information file, Table S1, entries 1–14). First of all, in the absence of designed magnetic nanocatalyst, only 20% reaction progression was observed at room temperature condition during 15 min (See the supplementary information file, Table S1, entry 1). Furthermore, the model reaction was run with a determined amount of magnetized rGO aerogel, HKUST-1 particles, and bare Fe_3_O_4_ MNPs (20 mg) at room temperature condition (See the supplementary information file, Table S1, entries 2-4). In the presence of 20 mg of Fe_3_O_4_ MNPs, the reaction progress was not successful (See the supplementary information file, Table S1, entry 4). However, interestingly, compared to the model reaction without using catalyst (See the supplementary information file, Table S1, entry 1), the reaction progression was considerably enhanced at the same time (15 min); therefore, it was deduced that the catalytic activity of HKUST-1 and magnetized rGO aerogel in the form of magnetic rGO aerogel/HKUST-1 nanocomposite would be more. In this step, given the room temperature condition (25 °C), 10 mg, 20 mg, and 30 mg of designed magnetic nanocatalyst and different types of polar and non-polar solvents were examined (See the supplementary information file, Table S1, entries 5-14). After processing the examinations, it was determined that 20 mg of designed magnetic nanocatalyst as an optimum amount could be accompanied by the highest yield percentage of product (92%) at room temperature condition and using green ethanol solvent (See the supplementary information file, Table S1, entry 6). Following optimizing the reaction condition, the catalytic study of magnetic rGO aerogel/HKUST-1 nanocomposite was conducted in the synthesis of polyhydroquinoline derivatives. In this step, a diversity of aromatic aldehydes (**1**) substituted with electron-donating and electron-withdrawing groups, ethyl acetoacetate (**2**), dimedone (**3**), and ammonium acetate (**4**) (See the supplementary information file, Table S2, entries 1-16) was used; and reasonable yield percentage of products were synthesized.

#### Mechanism study of magnetic rGO aerogel/HKUST-1 nanocomposite in the one-pot synthesis of polyhydroquinoline derivatives

**Figure 8:**
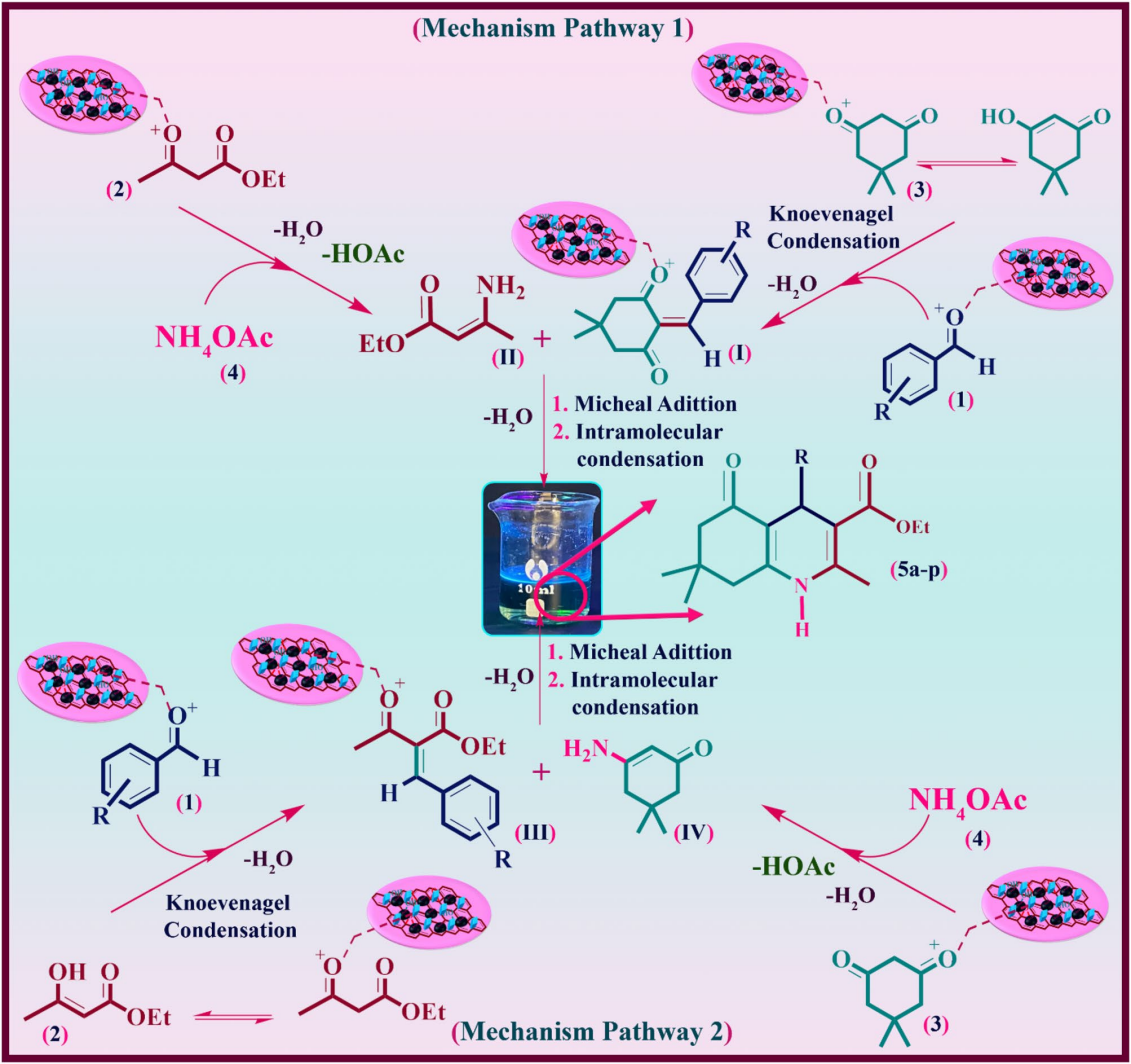
Proposed mechanism and catalytic activity of magnetic rGO aerogel/HKUST-1 nanocomposite in the synthesis of polyhydroquinoline derivatives.

Considering the great potential of graphene-based aerogels as an efficient catalytic support^9^ and the presence of unsaturated metal sites of HKUST-1 particles^46^ in catalytic applications, their combination with magnetic property is presented as a magnetic rGO aerogel/HKUST-1 nanocomposite, a new magnetic nanostructure with catalytic synergetic effect. The outline of suggested mechanism pathway is illustrated in Fig. 8. According to the reported research literature^47^, in order to synthesize polyhydroquinoline derivatives, two mechanism pathways can be proposed. As can be seen in the first mechanism pathway, first, the intermediate **I** is formed by conducting the Knoevenagel condensation reaction between the enol structure of activated dimedone (**3**) and activated substituted aldehyde (**1**). In the next step, a reaction between ammonium acetate (**4**) and activated ethyl acetoacetate (**2**) is performed to obtain the intermediate **II** with an enamine structure. Subsequently, after the Michael addition reaction between the intermediate **I** and intermediate **II**, the cyclization reaction and dehydration process are followed to yield polyhydroquinoline derivatives (**5a-p**). In the second mechanism pathway, given the Knoevenagel condensation reaction between the enol form of activated ethyl acetoacetate (**2**) and activated aldehyde (**1**), the intermediate **III** is generated. Afterwards, the reaction is conducted between the activated dimedone (**3**) and ammonium acetate (**4**) to generate intermediate **IV**. Following the Michael addition reaction between the two intermediates (**III, IV**), the synthesis process of polyhydroquinoline derivatives (**5a-p**) is completed by cyclization and dehydration reactions.

#### Comparing the catalytic activity of magnetic rGO aerogel/HKUST-1 nanocomposite in the one-pot synthesis of polyhydroquinoline derivatives with other studies

Considering various reaction conditions and suggesting a diversity of homogeneous and heterogeneous catalysts to synthesize polyhydroquinoline derivatives, the catalytic capacity of magnetic rGO aerogel/HKUST-1 nanocomposite was assayed with previous research studies (See the supplementary information file, Table S3, entries 1–10). In this context, apart from the model reaction including benzaldehyde, ethyl acetoacetate, dimedone, and ammonium acetate, the catalyst amount, solvent, temperature, reaction time, and yield percentage of product were investigated and the results were summed up. In comparison to reported catalytic research works (See the supplementary information file, Table S3, entries 1–9), applying magnetic aerogel/HKUST-1 nanocatalyst (See the supplementary information file, Table S3, entry 10) can lead to synthesize polyhydroquinoline compounds in a preferable and optimized reaction condition. Small amount of designed magnetic nanocatalyst with excellent catalytic performance is sufficient to synthesize these bioactive compounds in a shorter time, green reaction media (ethanol), and simple work-up process.

#### Optimization of reaction parameters in the one-pot synthesis of 1,8-dioxo-decahydroacridine derivatives

Apart from the catalytic performance of magnetic rGO aerogel/HKUST-1 nanocomposite to synthesize polyhydroquinoline derivatives, its catalytic activity was evaluated in one-pot three-condensation reaction of 1,8-dioxo-decahydroacridine derivatives. In this step, to optimize different reaction parameters (amount of catalyst, temperature, solvent), a model reaction mixture including benzaldehyde (0.4 mmol), dimedone (1 mmol), and ammonium acetate (2 mmol) was considered (See the supplementary information file, Table S4, entries 1–14). Like polyhydroquinoline compounds, it was hypothesized that 1,8-dioxo-decahydroacridine derivatives could be synthesized at room temperature condition; therefore, a test was conducted with 10 mg of magnetic nanocatalyst at 25 °C (See the supplementary information file, Table S4, entry 1). After 1 h, the reaction was not accompanied by any progression. Subsequently, the reflux condition (80 °C) without the nanocatalyst, and different amounts of nanocatalyst (10 mg, 20 mg, 30 mg) was examined in the ethanol solvent (See the supplementary information file, Table S4, entries 2,5–7). Given the new thermal condition, it was understood that the temperature increment could reduce the time of model reaction (10 min) and increase the yield percentage of product from 30% to 93% (See the supplementary information file, Table S4, entries 6–7). Alongside the temperature increment, the efficiency of model reaction was evaluated in different amounts of nanocatalyst (See the supplementary information file, Table S4, entries 5–7) and as well, the reaction process was analyzed in different solvents such as ethanol (reflux, 80 °C), distilled water (80 °C), toluene (80 °C), dimethylformamide (80 °C), methanol (reflux), chloroform (reflux), dichloromethane (reflux), and acetonitrile (reflux) (See the supplementary information file, Table S4, entries 6, 8–14). Subsequently, the highest yield percentage of product was observed in green ethanol with possible optimized amount of nanocatalyst (20 mg) (See the supplementary information file, Table S4, entry 6). In addition to this, it was appraised that higher yield percentage of product cannot be obtained using 20 mg of HKUST-1 or magnetized rGO aerogel alone (See the supplementary information file, Table S4, entries 3–4). In other words, the in-situ formation of HKUST-1 in the presence of this prepared magnetized substrate can be accompanied by a synergetic catalytic effect and further reaction progress in a short time. After optimizing the reaction condition, the catalytic generality of magnetic rGO aerogel/HKUST-1 nanocomposite was studied in the synthesis of 1,8-dioxo-decahydroacridine products. Several aromatic aldehydes (**1**) with electron-donating and withdrawing substitutions were reacted with dimedone (**2**), and ammonium acetate (**3**) (See the supplementary information file, Table S5, entries 1–10); and excellent yield percentage of products were fabricated.

#### Mechanism study of magnetic rGO aerogel/HKUST-1 nanocomposite in the one-pot synthesis of 1,8-dioxo-decahydroacridine derivatives

Alongside the synthesis process of polyhydroquinoline derivatives, the presence and functionality of magnetic rGO aerogel/HKUST-1 nanocomposite as a leading catalytic factor were assessed in the synthesis of 1,8-dioxo-decahydroacridine derivatives. The outline of suggested mechanism pathway is displayed in the supplementary information file, Fig. S8. Similarly, after the activation of carbonyl group of aldehydes (**1**) and dimedone (**2**) by designed magnetic nanocatalyst, the Knoevenagel condensation reaction is conducted between these two components (aldehyde, enol structure of dimedone) to form intermediate **I**. Following that, the intermediate **II** is generated by the reaction between the second enol structure of dimedone (**2**) and ammonium acetate (**3**). Eventually, after the formation of open chain intermediate **III** obtained from the Michael addition reaction between the intermediate **I** and intermediate **II**, given the consecutive cyclization and dehydration reactions, the desired 1,8-dioxo-decahydroacridines (**6a-j**) are synthesized.

#### Comparing the catalytic activity of magnetic rGO aerogel/HKUST-1 nanocomposite with other studies

According to the synthesis process of 1,8-dioxo-decahydroacridine derivatives using different catalysts, and chemical reaction conditions, the catalytic performance of magnetic rGO aerogel/HKUST-1 nanocomposite was compared with other research studies (See the supplementary information file, Table S6, entries 1–10). For this purpose, the model reaction including benzaldehyde, dimedone, ammonium acetate and also, the catalyst amount, solvent, temperature, reaction time, and yield percentage of product was considered. As a detailed assessment (See the supplementary information file, Table S6, entries 1–9), and compared to the homogenous and heterogeneous catalytic studies, it was deduced that 1,8-dioxo-decahydroacridine derivatives can be synthesized in a better and optimized reaction condition by using magnetic rGO aerogel/HKUST-1 nanocatalyst (See the supplementary information file, Table S6, entry 10). By using a small amount of designed magnetic nanostructure owning high catalytic activity and magnetic property, and as well, ease of work-up process, the synthesis process can be conducted in a shorter time, and green reaction media.

#### Catalytic recyclability of magnetic rGO aerogel/HKUST-1 nanocomposite

Given the synthesis protocols and green chemistry perspectives, two predominant factors including the catalytic recovery, and recyclability of nanocatalysts must be considered. The catalytic recyclability of magnetic rGO aerogel/HKUST-1 nanocomposite (20 mg) was evaluated in the synthesis of polyhydroquinoline, and 1,8-dioxo-decahydroacridine derivatives five times. For this purpose, after each catalytic run and reaction completion, first, the magnetic nanocatalyst was separated using an external neodymium magnet. Next, the separated nanocatalyst was eluted with ethanol five times and then, it was dried in the vacuum oven (80 °C, 24 h) to be prepared for the subsequent catalytic run. As indicated in the supplementary information file, Fig. S9a-b, after reusing a constant amount of dried nanocatalyst, and due to the results obtained from each optimized reaction, it can be mentioned that the magnetic rGO aerogel/HKUST-1 nanocatalyst can be easily recycled at least five runs without any significant reduction in isolated yield percentage of product. In addition to this, the structural stability of recycled magnetic rGO aerogel/HKUST-1 nanocatalyst was confirmed by using FT-IR analysis, SEM imaging, and XRD Pattern (See the supplementary information file, Figs. S10-S12).

## Conclusions

As summary, in this research study, given the importance of graphene-based aerogel and extensive functionality of metal-organic frameworks, new magnetic rGO aerogel/HKUST-1 nanocomposite was designed and synthesized according to the transformation of graphene oxide sheets to three-dimensional reduced graphene oxide aerogel, the in-situ magnetization of aerogel substrate, and the in-situ formation of HKUST-1 particles. In terms of chemical structure, several spectral and analytical techniques including FT-IR, EDX, ICP, FE-SEM, DLS, XRD, VSM, and TG analyses were taken and the results were entirely assessed. Furthermore, the catalytic performance and capacity of this new magnetic nanocomposite were examined in a one-pot synthesis of biologically active polyhydroquinoline and 1,8-dioxo-decahydroacridine derivatives. Taking into account the results obtained from the reactions, it was deduced that the composition of magnetized reduced graphene oxide aerogel and HKUST-1 particles in the form magnetic rGO aerogel/HKUST-1 nanocomposite with small amount and having high synergetic catalytic effect can run the symmetric and unsymmetrical Hantzsch condensation reactions in a shorter reaction time, ease of work-up process, and yielding high percentage of product.

## Experimental section

### General

In this research study, all of the chemical reagents and solvents with analytical grade and high purity were poured in advance from Sigma-Aldrich company. An external neodymium magnet with NdFeB material, a determined dimension (50 mm × 40 mm × 20 mm), a rectangular cube block shape (298 g), maximum tensile strength (75 kg), and maximum slip resistance (25 kg) was used to synthesize the magnetic rGO aerogel/HKUST-1 nanocomposite and separate it from the reaction media. The melting point of all solid isolated derivatives obtained, were recorded using electrothermal 9100 apparatus. ^1^H NMR and ^13^C NMR of isolated derivatives were determined by Avance spectrometer (Bruker-400 model) at 400 and 100 MHz respectively. To characterize and evaluate the chemical structure of magnetic rGO aerogel/HKUST-1 nanocomposite, several analytical and spectral analyses were performed. The formation of new functional groups was confirmed using Fourier-transform infrared (FT-IR) spectrometer (Perkin Elmer,1720-X model, USA) with the method of KBr pellet. The elemental composition from each synthesis step was characterized; and as well, the distribution pattern images of structural elements related to the magnetic rGO aerogel/HKUST-1 nanocomposite were taken by energy-dispersive X-ray (EDX) analysis (Oxford instrument, England) using gold sputtering coating technique (Agar Sputter Coater model, Agar Scientific, England). The weight percentage of copper in the structure of designed magnetic nanocomposite was determined by inductively coupled plasma optical emission spectrometer (ICP-OES) (SPECTRO, ARCOS, Germany). The structure, morphology, and the X-ray diffraction (XRD) pattern were recorded using field-emission scanning microscope (FE-SEM) (ZEISS-Sigma VP model, Germany) and Brucker X-ray diffractometer device (D8 Advanced model, USA) from each synthesis step. Furthermore, scanning electron microscope (SEM) (TESCAN VEGA II xmu model, Czech Republic) was used to evaluate the structure of reusable magnetic catalyst. Dynamic light scattering (DLS) analysis (Nano-flex, 180°, DLS size model, Particle Metrix company, Germany) was taken to characterize the particle size distribution of bare Fe_3_O_4_ MNPs and HKUST-1 particles. To compare the magnetic property of bare Fe_3_O_4_ MNPs, magnetized rGO aerogel, and designed magnetic rGO aerogel/HKUST-1 nanocomposite, their hysteresis loop curves were recorded using vibrating-sample magnetometer (VSM) (LBKFB model magnetic kavir, − 10,000 Oe to + 10,000 Oe, Iran). Furthermore, the thermogravimetric behaviour was analysed using thermogravimetric analyzer instrument (TGA2 model, Mettler Toledo, USA). Besides, each thermal cycle was run between 25 ^o^C to 600 °C with a constant heating rate (10 °C /min).

### Preparation of graphene oxide

The synthesis process of GO was performed according to the reported modified Hummer methods^48,49^. First, in a beaker 23 mL of sulfuric acid (98%) was added to a beaker (1000 mL) filled with 1.0 g of natural purified graphite, and then, followed by adding 0.5 g of sodium nitrate, the suspension solution was continuously stirred at 66 °C for 20 min. Next, to disperse and homogenize the suspension solution, the beaker was sonicated in an ultrasonic bath (25 °C) for 30 min. Following the ultrasonic bath condition and decreasing the temperature less than 20 °C, 3.0 g of potassium permanganate was gradually added to the reaction mixture due to the exothermic reaction mixture. After adding potassium permanganate, the suspension mixture was homogenized using the stirring condition (30 °C) for 30 min. Next, 50 mL of distilled water was slowly added to the suspension solution in a continuous stirring condition. Keeping the suspension reaction stirred, the reaction temperature was increased up to 98 °C and as well, the stirring condition (98 °C) was continued for a further 30 min. After that, a yellow–brown solution was obtained by adding 700 mL of distilled water and 12 mL of hydrogen peroxide. Then, 2% hydrochloric acid solution (2 mL HCl in 98 mL of distilled water) was added to adjust the pH of solution. Eventually, the mixture solution was filtered and washed with distilled water three times; and followed by oak brown powder dried in the vacuum oven (60 °C) for 24 h.

### Preparation of three-dimensional reduced graphene oxide aerogel

As reported in previous research studies^50^, the synthesis process of three-dimensional rGO aerogel was conducted using the hydrothermal condition. In a typical experiment, first, 40 µL of ammonia solution (25%) was added to 10 mg of dispersed GO solution (3 mg mL^−1^) and the mixture solution was kept in the sonication condition (ultrasonic bath, 25 °C) for 1 h. Following that, the mixture solution was transferred to a Teflon-lined autoclave and the reaction was heated at 180 °C for 24 h. After the mentioned time, the autoclave reactor was cooled down to room temperature. To remove unreacted reagents and impurities, the prepared three-dimensional rGO hydrogel was eluted with distilled water several times, and subsequently, it was kept at freezer (-70 °C) for 24 h. Finally, to obtain three-dimensional rGO aerogel, the solvent sublimation was performed using the freeze-dryer device in a constant temperature and pressure condition (-60 °C, 0.1 bar) for 24 h.

### Magnetization process of reduced graphene oxide aerogel

The magnetization process of rGO aerogel was conducted according to the following steps. First, 2.91 g of FeCl_3_.6H_2_O and 1.33 g of FeCl_2_.4H_2_O salts were dissolved in 40 mL of distilled water. Then, 0.5 g of prepared rGO aerogel was added to the mixture solution and it was stirred at 70 °C and N_2_ atmosphere for 30 min. Next, 25 mL of 25% aqueous ammonia was drop wisely injected to the reaction solution in 30 min. Followed by the addition of aqueous ammonia, the reaction solution was continuously stirred at 70 °C for 2 h. Afterward, the reaction solution was cooled down at room temperature. The obtained black precipitate was separated using an external neodymium magnet, and washed with distilled water (several times) to eliminate the unreacted reagents and reach a neutral pH value (pH = 7). The prepared magnetized rGO aerogel nanostructure was dried at 60 °C for 24 h.

### Preparation of magnetic rGO aerogel/HKUST-1 nanocomposite

To synthesize magnetic rGO aerogel/HKUST-1 nanocomposite, given the reported hydrothermal protocol of HKUST-1 metal-organic framework^51^, with slight modifications, initially, 0.875 g of Cu(NO_3_)_2_.3H_2_O was dissolved in 15 mL of distilled water. 1 g of magnetic rGO aerogel was gradually added to the solution and dispersed well under the stirring condition for 30 min. Then, the prepared trimesic acid solution (0.42 g in 12 mL of ethanol) was drop wisely added to the mixture solution and stirred at room temperature condition for 1 h. After the relevant time, the reaction mixture was transferred to a Teflon-lined autoclave and it was heated at 120 °C for 24 h. After cooling down the autoclave reactor, the magnetic rGO aerogel/HKUST-1 nanocomposite obtained from the reaction washed with distilled water, ethanol, and it was dried in the vacuum oven (80 °C) for 24 h.

### General procedure for one-pot synthesis of polyhydroquinoline derivatives *(5a-q)*

Magnetic rGO aerogel/HKUST-1 nanocomposite as new nanocatalyst was evaluated for one-pot synthesis of polyhydroquinoline derivatives. A reaction media in ethanol solvent (4 mL) including several aromatic aldehydes with electron donating and withdrawing substitutions (0.4 mmol), dimedone (0.5 mmol), ethyl acetoacetate (0.5 mmol), ammonium acetate (2 mmol), and 20 mg of magnetic rGO aerogel/HKUST-1 nanocatalyst was stirred at room temperature condition. The reaction progress was monitored by thin layer chromatography method (1:3). After the reaction completion, first, the magnetic nanocatalyst was separated using an external neodymium magnet. Then, the reaction mixture was evaporated under reduced pressure, washed with water, and then, the recrystallization process was performed in ethanol to purify and obtain an isolated product.

### General procedure for one-pot synthesis of 1,8-dioxo-decahydroacridine derivatives *(6a-j)*

Apart from polyhydroquinoline derivatives, the catalytic efficiency of magnetic rGO aerogel/HKUST-1 nanocomposite was examined in one-pot synthesis of 1,8-dioxo-decahydroacridine derivatives too. A reaction media in ethanol solvent (4 mL) including several aromatic aldehydes with electron donating and withdrawing substitutions (0.4 mmol), dimedone (1 mmol), ammonium acetate (2 mmol), and 20 mg of designed magnetic nanocatalyst was stirred at reflux condition (80 °C). The reaction progress was monitored by thin layer chromatography method (1:2). After the completion of reaction, first, the magnetic nanocatalyst was separated using an external neodymium magnet. Next, the reaction mixture was evaporated under reduced pressure, washed with water, and then, the recrystallization process was performed in ethanol to purify and obtain an isolated product.

### Spectral data of isolated derivative products

Ethyl 2,7,7-trimethyl-5-oxo-4-phenyl-1,4,5,6,7,8- hexahydroquinoline-3-carboxylate **(5a).**

^1^H NMR (400 MHz, DMSO): *δ*_H_ (ppm) = 0.84 (s, 3H), 1.01 (s, 3H), 1.13 (t, J = 8 Hz, 3H), 1.97 (d, J = 20 Hz, 1H), 2.17 (d, J = 16 Hz, 1H), 2.27–2.31 (m with s at 2.27, 4H), 2.42 (d, J = 16 Hz, 1H), 3.97 (q, J = 8 Hz, 2H), 4.85 (s, 1H), 7.05–7.09 (m, Ar–H, para-H), 7.14–7.18 (m, Ar–H, 4H), 9.07 (br. s, 1H, N–H); ^13^C NMR (100 MHz, DMSO); *δ*_C_ (ppm) = 194.72, 167.31, 149.98, 148.10, 145. 46, 128.20, 127.92, 126.15, 110.41, 104.04, 59.50, 50.70, 36.30, 32.62, 29.61, 26.92, 18.76, 14.62.

Ethyl 4-(2-chlorophenyl)-2,7,7-trimethyl-5-oxo1,4,5,6,7,8-hexahydroquinoline-3-carboxylate **(5h).**

^1^H NMR (400 MHz, DMSO): *δ*_H_ (ppm) = 0.84 (s, 3H), 1.01 (s, 3H), 1.08 (t, J = 8, 3H), 1.89–1.93 (d, 1H), 2.12–2.16 (d, 1H), 2.25–2.28 (m with s at 2.25, 4H), 2.40–2.45 (d, 1H), 3.87–3.97 (m, 2H), 5.18 (s. 1H), 7.05–7.18 (m, 1H), 7.18–7.21 (m, 2H), 7.21–7.29 (m, 1H), 9.10 (br. s, 1H, N–H); ^13^C NMR (100 MHz, DMSO); *δ*_C_ (ppm) = 194.34, 167.24, 150.17, 145.48, 132.33, 131.95, 129.44, 127.74, 127.13, 110.02, 103.69, 59.42, 50.70, 35.32, 32.46, 29.59, 26.85, 18.66, 14.58.

### Supplementary Information

Supplementary Information.

## Data Availability

The datasets used and/or analysed during the current study available from the corresponding author on reasonable request.
