# Supplementary material for: Development of new magnetic nanocomposite designed by reduced graphene oxide aerogel and HKUST-1, and its catalytic application in the synthesis of polyhydroquinoline and 1,8-dioxo-decahydroacridine derivatives
Source: Sci Rep. 2023 Dec 21;13:22913. doi: 10.1038/s41598-023-48674-5 (PMC10739897; doi:10.1038/s41598-023-48674-5)
Supplement: Supplementary file 1 — Supplementary Information. [file 41598_2023_48674_MOESM1_ESM.docx]

**Supplementary information**

# Development of new magnetic nanocomposite designed by reduced graphene oxide aerogel and HKUST-1, and its catalytic application in the synthesis of polyhydroquinoline and 1,8-dioxo-decahydroacridine derivatives

Naghmeh Farzaneh^1,2^, Fateme Radinekiyan^1,†^, Mohammad Reza Naimi-Jamal^1^*, Mohammad G. Dekamin^2^

^1^Research Laboratory of Green Organic Synthesis and Polymers, Department of Chemistry, Iran University of Science and Technology, P.O. Box 16846‑13114, Tehran, Iran.

^2^Pharmaceutical and Heterocyclic Compounds Research Laboratory, Department of Chemistry, Iran University of Science and Technology, Tehran, 16846-13114, Iran.

^†^This author contributed equally: Fateme Radinekiyan

*Corresponding authors. (M. R. Naimi-Jamal)

| Table of contents | | |
| --- | --- | --- |
| Entry | **Subject** | **Page** |
| 1 | **Data characterization of bare Fe_3_O_4_ MNPs and HKUST-1 particles** | (S3) |
| 2 | Fig. S1. Particle size distribution graphs of (a) bare Fe_3_O_4_ MNPs, (b) HKUST-1 particles | S3 |
| 1 | **Data characterization of polyhydroquinoline and 1,8-dioxo-decahydroacridine derivatives derivatives** | (S4-S9) |
| 2 | Fig. S2. FT-IR spectrum of product **(5a)**. | S4 |
| 3 | Fig. S3_._ ^1^H NMR of product **(5a)**. | S5 |
| 4 | Fig. S4. ^13^C NMR of product **(5a)**. | S6 |
| 5 | Fig. S5. FT-IR spectrum of product **(5h)**. | S7 |
| 6 | Fig. S6_._ ^1^H NMR of product **(5h)**. | S8 |
| 7 | Fig. S7. ^13^C NMR of product **(5h)**. | S9 |
| 8 | Table S1. Optimization of different reaction parameters given the model reaction^a^. | S10 |
| 9 | Table S2. One-pot synthesis of polyhydroquinoline derivatives catalyzed by magnetic rGO aerogel/HKUST-1 nanocomposite. | S11-S13 |
| 10 | Table S3. Comparing the catalytic activity of magnetic rGO aerogel/HKUST-1 nanocomposite with other studies. | S14 |
| 11 | Table S4. Optimization of different reaction parameters given the model reaction.^a^. | S15 |
| 12 | Table S5. One-pot synthesis of 1,8-dioxo-decahydroacridine derivatives catalyzed by magnetic rGO aerogel/HKUST-1 nanocomposite. | S16-S17 |
| 13 | Fig. S8. Proposed mechanism and catalytic activity of magnetic rGO aerogel/HKUST-1 nanocomposite in the synthesis of 1,8-dioxo-decahydroacridine derivatives. | S18 |
| 14 | Table S6. Comparing the catalytic activity of magnetic rGO aerogel/HKUST-1 nanocomposite with other studies. | S19 |
| 15 | **Data characterization of recycled magnetic nanocatalyst** | (S20-S23) |
| 17 | Fig. S9. Catalytic recyclability line graph of magnetic rGO aerogel/HKUST-1 nanocomposite (20 mg) in a) the synthesis of product **5a** and b) the synthesis of product **6a**. | S20 |
| 1 | Fig. S10. FT-IR spectra of (a) magnetic rGO aerogel/HKUST-1 nanocatalyst, (b) recycled magnetic nanocatalyst. | S21 |
| 18 | Fig. S11. SEM images of (a-b) recycled magnetic rGO aerogel/HKUST-1 nanocatalyst. | S22 |
| 19 | Fig. S12. XRD patterns of (a) magnetic rGO aerogel/HKUST-1 nanocatalyst, (b) recycled magnetic nanocatalyst. | S23 |
| 20 | **Supplementary information file's references** | S24-S28 |

**Data characterization of bare Fe_3_O_4_ MNPs and HKUST-1 particles**

**
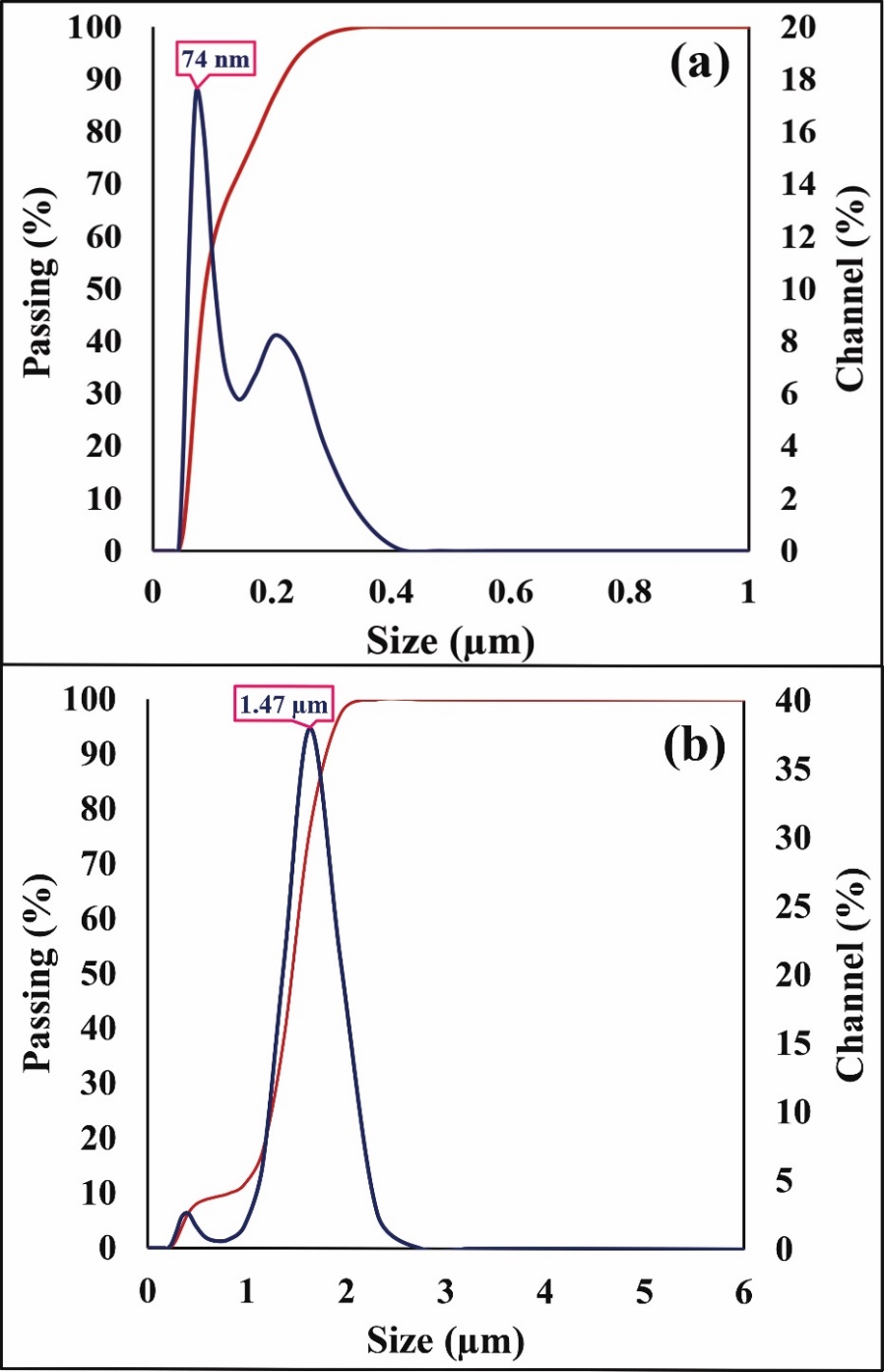
**

**Fig. S1.** Particle size distribution graphs of (a) bare Fe_3_O_4_ MNPs, (b) HKUST-1 particles.

**Data characterization of polyhydroquinoline derivatives**

**
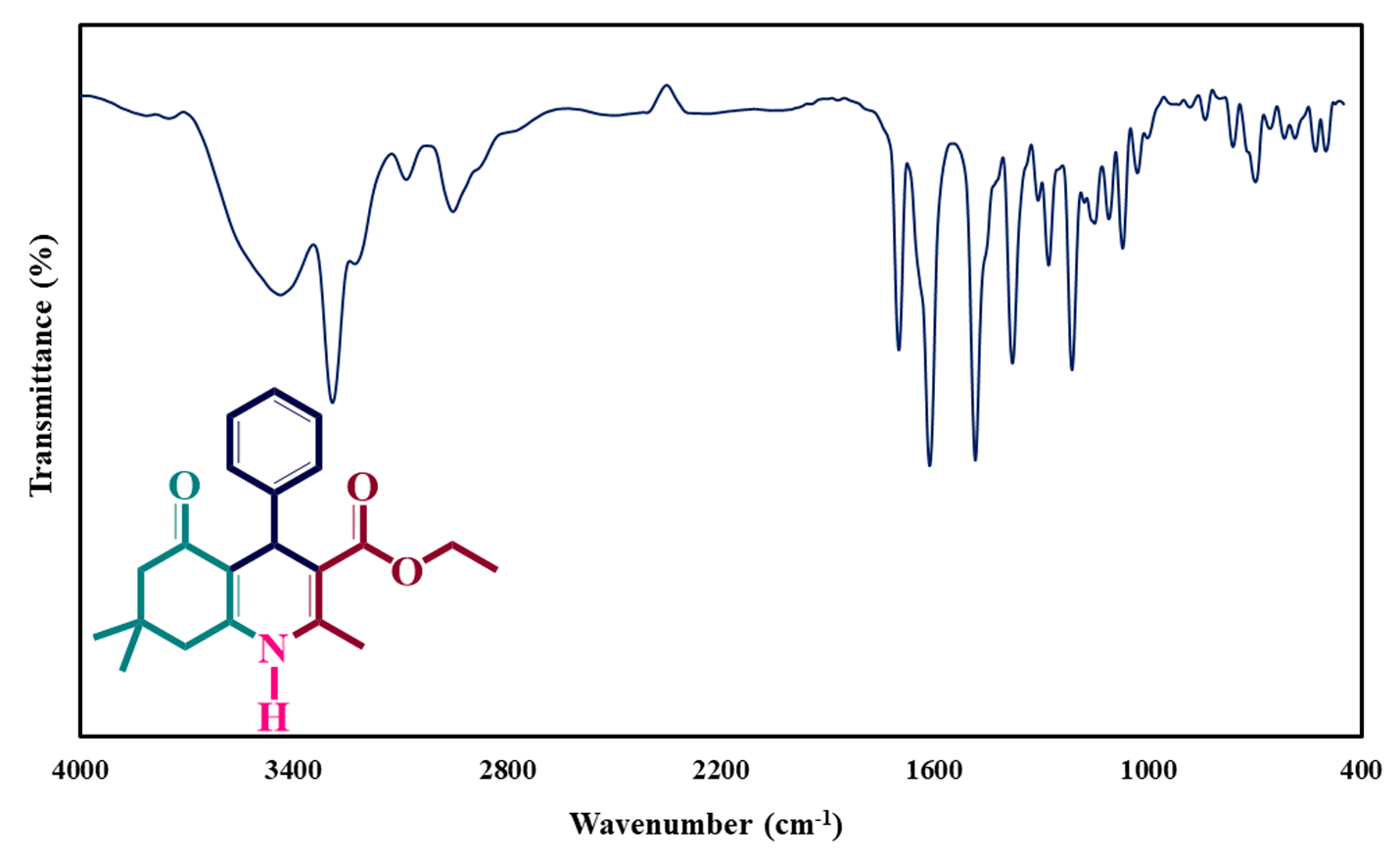
Fig. S2.** FT-IR spectrum of product **(5a)**.

**
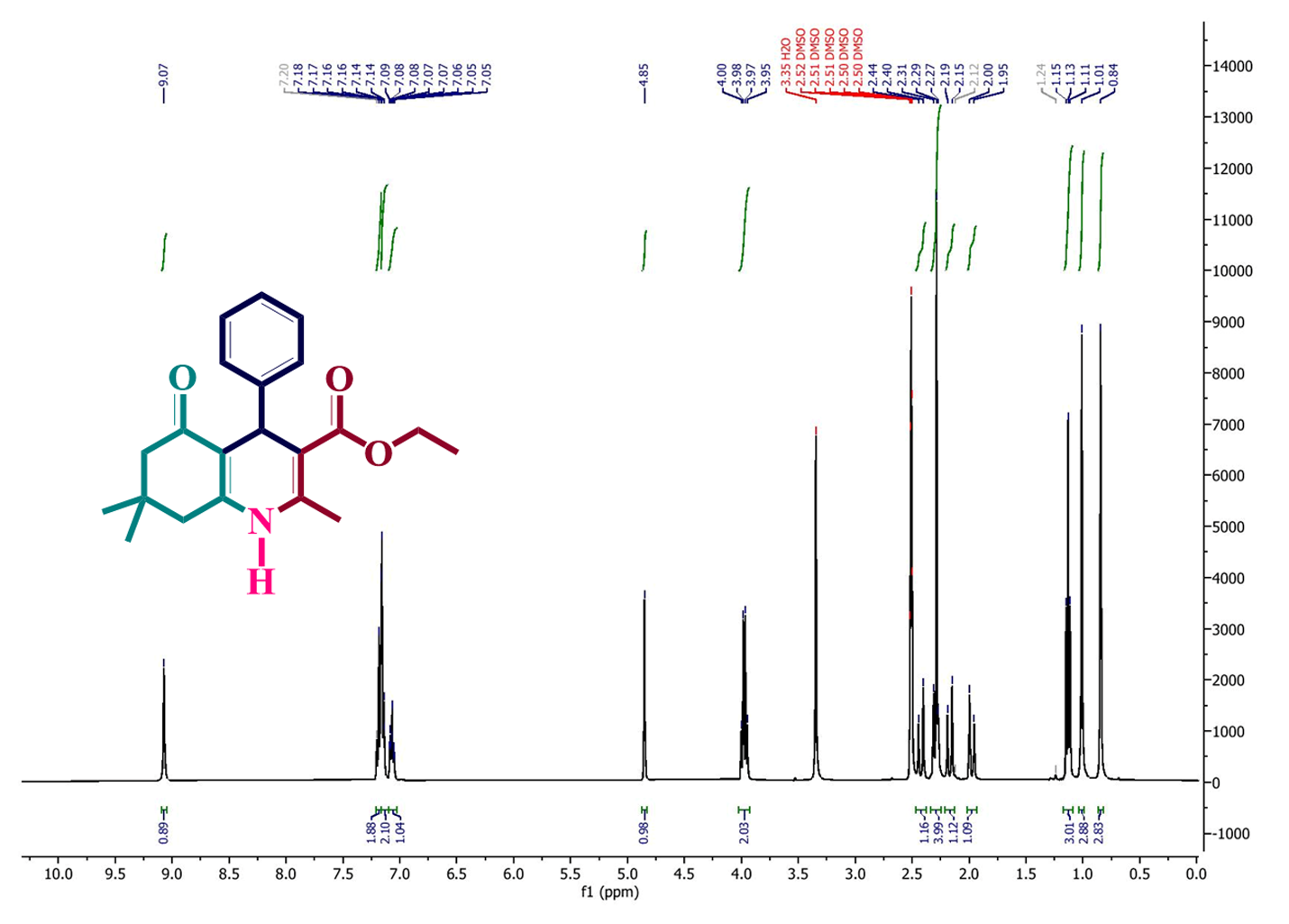
**

**Fig. S3.** ^1^H NMR spectrum of the product **(5a)**.


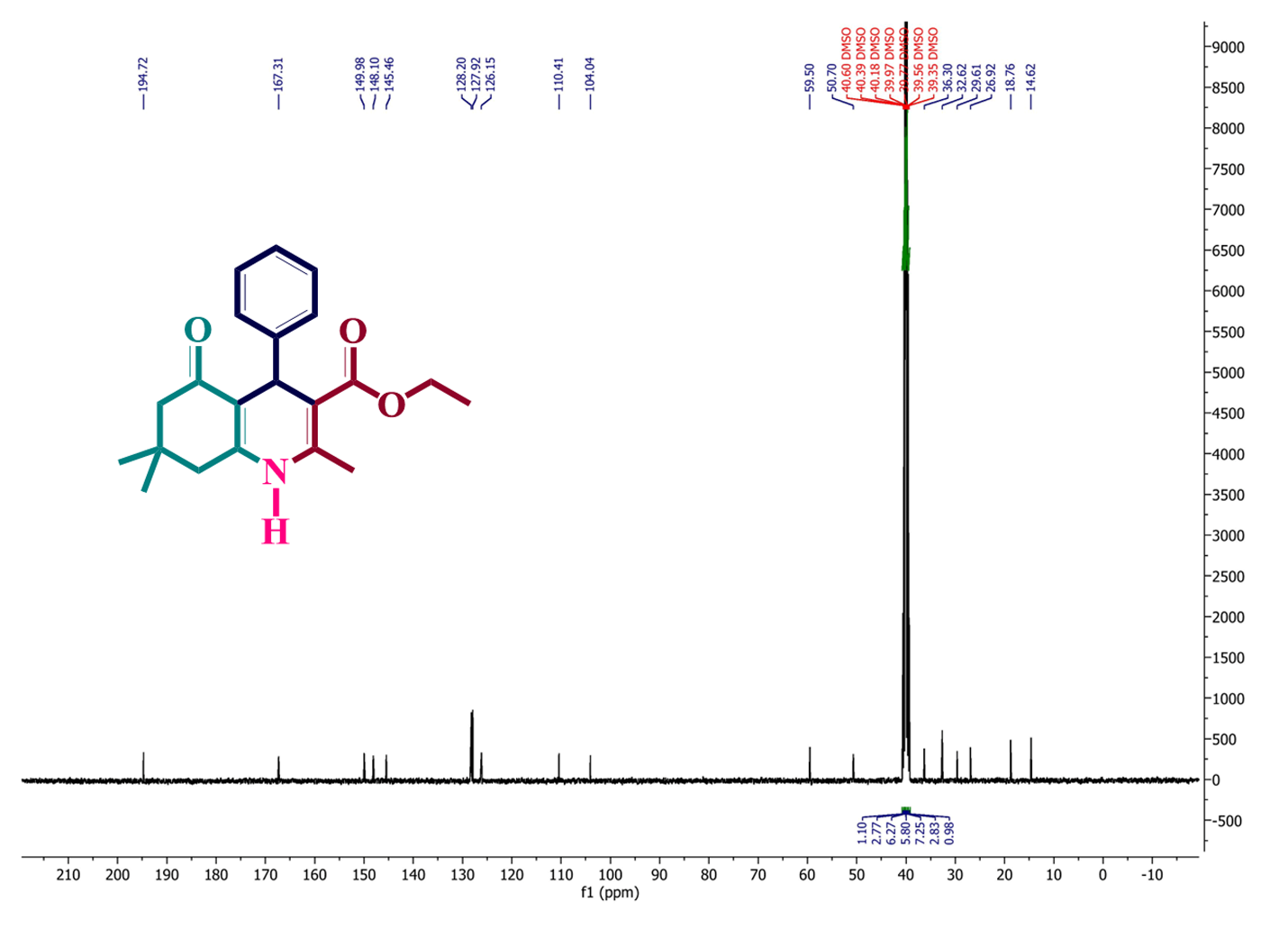


**Fig. S4.** ^1^C NMR spectrum of the product **(5a)**.

**
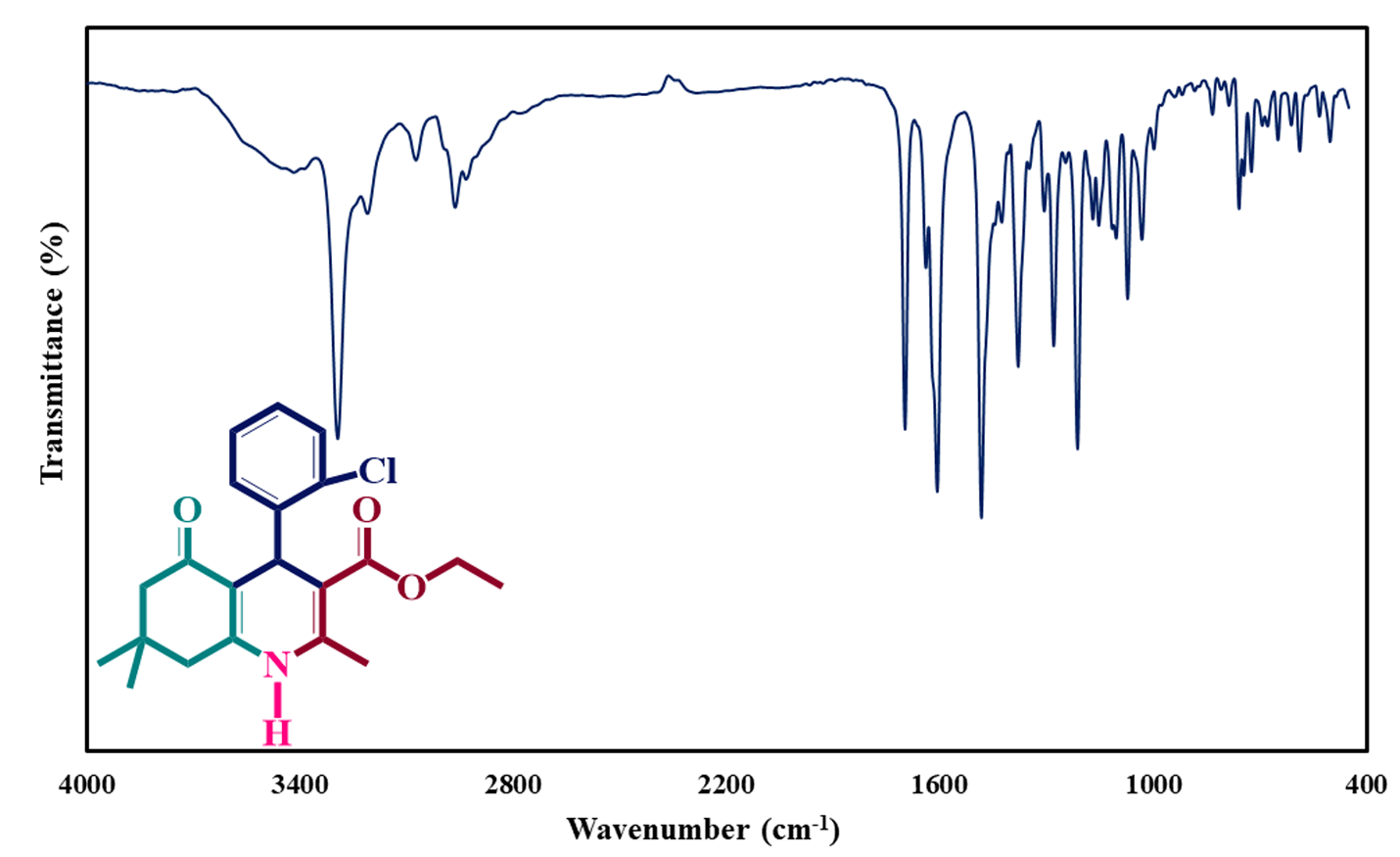
**

**Fig. S5.** FT-IR spectrum of product **(5h)**.

**
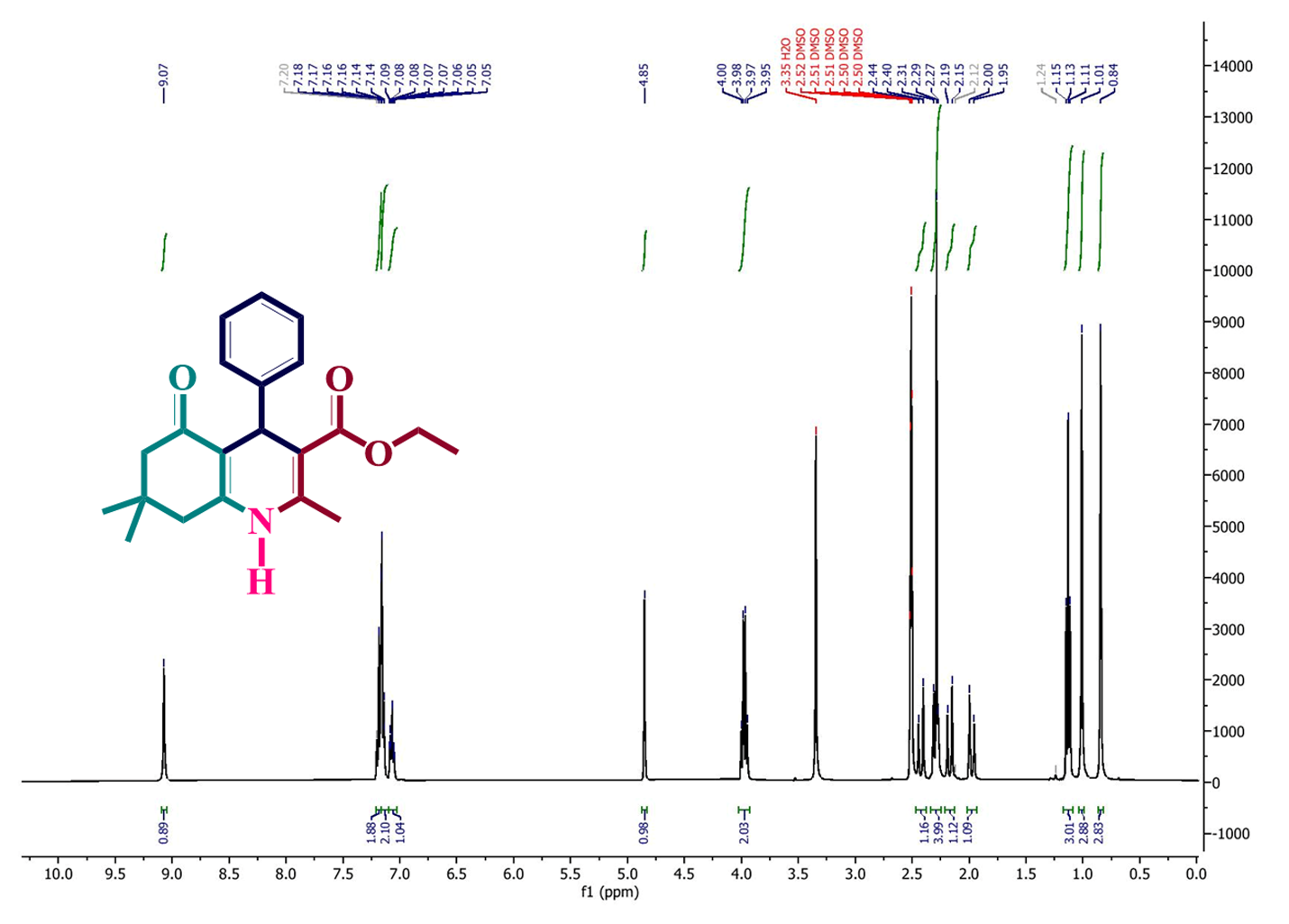
**

**Fig. S6.** ^1^H NMR of product **(5h)**.


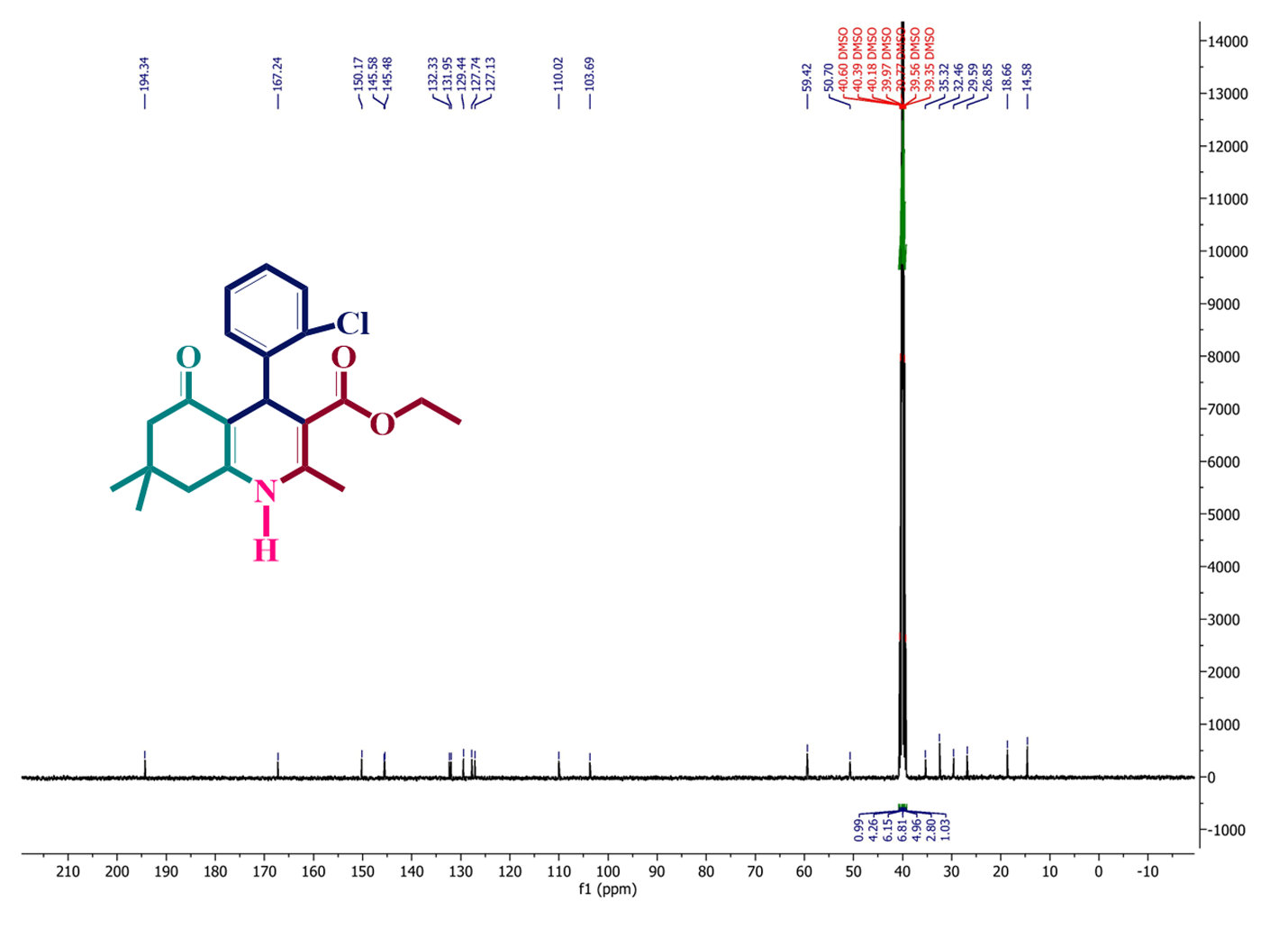


**Fig. S7.** ^13^C NMR of product **(5h)**.

**Table S1.** Optimization of different reaction parameters given the model reaction^a^.

| Entry | Catalyst (mg) | Solvent | Yield^b^ (%) |
| --- | --- | --- | --- |
| 1 | --------------- | EtOH | 20 |
| 2 | HKUST-1 (20 mg) | EtOH | 60 |
| 3 | RGO/Fe_3_O_4_ (20 mg) | EtOH | 47 |
| 4 | Fe_3_O_4_ (20 mg) | EtOH | 25 |
| 5 | Magnetic rGO aerogel/HKUST-1 nanocomposite (10 mg) | EtOH | 87 |
| 6 | Magnetic rGO aerogel/HKUST-1 nanocomposite (20 mg) | EtOH | 92 |
| 7 | Magnetic rGO aerogel/HKUST-1 nanocomposite (30 mg) | EtOH | 92 |
| 8 | Magnetic rGO aerogel/HKUST-1 nanocomposite (20 mg) | Toluene | 22 |
| 9 | Magnetic rGO aerogel/HKUST-1 nanocomposite (20 mg) | CH_3_CN | 50 |
| 10 | Magnetic rGO aerogel/HKUST-1 nanocomposite ((20 mg) | DMF | Trace |
| 11 | Magnetic rGO aerogel/HKUST-1 nanocomposite ((20 mg) | CH_2_Cl_2_ | 22 |
| 12 | Magnetic rGO aerogel/HKUST-1 nanocomposite (20 mg) | H_2_O | No reaction |
| 13 | Magnetic rGO aerogel/HKUST-1 nanocomposite (20 mg) | CHCl_3_ | No reaction |
| 14 | Magnetic rGO aerogel/HKUST-1 nanocomposite (20 mg) | MeOH | 85 |

^a^ Model reaction condition: benzaldehyde (0.4 mmol), ethyl acetoacetate (0.5 mmol), dimedone (0.5 mmol), ammonium acetate (2 mmol), room temperature (25 °C) in 15 min.

^b^ Isolated yield.

**Table S2.** One-pot synthesis of polyhydroquinoline derivatives^a^ catalyzed by magnetic rGO aerogel/HKUST-1 nanocomposite.

|  | | | | | | |
| --- | --- | --- | --- | --- | --- | --- |
| Entry | **R** | **Product** | **Time (min)** | **Yield^b^ (%)** | **Melting point (°C)**  **Observed** | **Melting point (°C)**  **Reported** |
| 1 |  |  | 15 | 92 | 218-220 | 218-220^1^ |
| 2 |  |  | 8 | 90 | 179-181 | 178-180^2^ |
| 3 |  |  | 10 | 88 | 240-242 | 240-242^3^ |
| 4 |  |  | 10 | 90 | 248-250 | 248-250^4^ |
| 5 |  |  | 15 | 90 | 258-260 | 258-260^5^ |
| 6 |  |  | 20 | 96 | 248-250 | 248-250^6^ |
| 7 |  |  | 20 | 95 | 228-230 | 228-230^7^ |
| 8 |  |  | 20 | 93 | 200-202 | 200-204^8^ |
| 9 |  |  | 25 | 93 | 240-242 | 240-242^9^ |
| 10 |  |  | 25 | 92 | 245-247 | 244-246^10^ |
| 11 |  |  | 20 | 80 | 240-242 | 240-242^11^ |
| 12 |  |  | 20 | 85 | 237-239 | 238-240^12^ |
| 13 |  |  | 30 | 89 | 228-230 | 228-230^13^ |
| 14 |  |  | 20 | 94 | 184-186 | 184-186^14^ |
| 15 |  |  | 15 | 94 | 238-240 | 238-240^15^ |
| 16 |  |  | 15 | 93 | 246-248 | 245-247^16^ |

^a^ Reaction conditions: (1) substituted aldehyde (0.4 mmol), (2) ethyl acetoacetate (0.5 mmol), (3) dimedone (0.5 mmol), (4) ammonium acetate (2 mmol), ethanol (4 mL), magnetic nanocatalyst (20 mg), room temperature.

^b^ Isolated yield.

**Table S3.** Comparing the catalytic activity of magnetic rGO aerogel/HKUST-1 nanocomposite with other studies^a^.

| Entry | Catalyst | Catalyst amount | Solvent | Condition / Temperature (°C) | Time (h:min) | Yield^b^ (%) | Ref |
| --- | --- | --- | --- | --- | --- | --- | --- |
| 1 | AFGONs | 25 mg | EtOH | R.T. | 2:00 | 90 | ^17^ |
| 2 | Yb(OTf)_3_ | 5 mol% | EtOH | R.T. | 5:00 | 90 | ^18^ |
| 3 | L-poline | 10 mol% | EtOH | Reflux | 6:00 | 92 | ^19^ |
| 4 | CTAB | 10 mol% | H_2_O | Reflux | 1:30 | 85 | ^22^ |
| 5 | Bakers yeast | 200 mg | Phosphate buffer | R.T. | 24:00 | 79 | ^23^ |
| 6 | Palladium NPs | 0.04 mmol | THF | Reflux | 4:00 | 89 | ^24^ |
| 7 | GSA@Fe_3_O_4_ MNPs | 50 mg | EtOH | Reflux / (80 °C) | 4:00 | 90 | ^25^ |
| 8 | CoFe_2_O_4_@Pr | 40 mg | EtOH | Reflux | 6:40 | 91 | ^26^ |
| 9 | CAN | 50 mg | EtOH | R.T. | 1:00 | 92 | ^27^ |
| 10 | Magnetic rGO aerogel/HKUST-1 nanocomposite | 20 mg | EtOH | R.T. | 15 min | 92 | Present study |

^a^ Reaction mixture: benzaldehyde (0.4 mmol), ethyl acetoacetate (0.5 mmol), dimedone (0.5 mmol), ammonium acetate (2 mmol).

^b^ Isolated yield.

**Table S4.** Optimization of different reaction parameters given the model reaction^a^.

| Entry | Catalyst (mg) | Solvent | Condition / Temperature (°C) | Time (min) | Yield^b^  (%) |
| --- | --- | --- | --- | --- | --- |
| 1 | Magnetic rGO aerogel/HKUST-1 nanocomposite  10 mg | EtOH | R.T. / (25 °C) | 60 | No reaction |
| 2 | -------- | EtOH | Reflux / (80 °C) | 10 | 30 |
| 3 | HKUST-1 (20 mg) | EtOH | Reflux / (80 °C) | 10 | 70 |
| 4 | Magnetized rGO aerogel (20 mg) | EtOH | Reflux / (80 °C) | 10 | 65 |
| 5 | Fe_3_O_4_ MNPs (20 mg) | EtOH | Reflux / (80 °C) | 10 | 36 |
| 6 | Magnetic rGO aerogel/HKUST-1 nanocomposite  10 mg | EtOH | Reflux / (80 °C) | 10 | 90 |
| 7 | Magnetic rGO aerogel/HKUST-1 nanocomposite  20 mg | EtOH | Reflux / (80 °C) | 10 | 93 |
| 8 | Magnetic rGO aerogel/HKUST-1 nanocomposite  30 mg | EtOH | Reflux / (80 °C) | 10 | 93 |
| 9 | Magnetic rGO aerogel/HKUST-1 nanocomposite  20 mg | Toluene | (80 °C) | 10 | 40 |
| 10 | Magnetic rGO aerogel/HKUST-1 nanocomposite  20 mg | CH_3_CN | Reflux | 10 | 60 |
| 11 | Magnetic rGO aerogel/HKUST-1 nanocomposite  20 mg | DMF | (80 °C) | 10 | 60 |
| 12 | Magnetic rGO aerogel/HKUST-1 nanocomposite  20 mg | CH_2_Cl_2_ | Reflux | 10 | Trace |
| 13 | Magnetic rGO aerogel/HKUST-1 nanocomposite  20 mg | H_2_O | (80 °C) | 10 | No reaction |
| 14 | Magnetic rGO aerogel/HKUST-1 nanocomposite  20 mg | CHCl_3_ | Reflux | 10 | No reaction |
| 15 | Magnetic rGO aerogel/HKUST-1 nanocomposite  20 mg | MeOH | Reflux | 10 | 83 |

^a^ Model reaction condition: benzaldehyde (0.4 mmol), dimedone (1 mmol), ammonium acetate (2 mmol).

^b^ Isolated yield.

**Table S5.** One-pot synthesis of 1,8-dioxo-decahydroacridine derivatives^a^ catalyzed by magnetic rGO aerogel/HKUST-1 nanocomposite.

|  | | | | | | |
| --- | --- | --- | --- | --- | --- | --- |
| Entry | **R** | **Product** | **Time (min)** | **Yield^b^(%)** | **Melting point (°C)**  **Observed** | **Melting point (°C)**  **Reported** |
| 1 |  |  | 10 | 93 | 250-252 | 250-252^28^ |
| 2 |  |  | 5 | 92 | 259-261 | 258-261^29^ |
| 3 |  |  | 10 | 88 | 275-277 | 275-277^30^ |
| 4 |  |  | 10 | 87 | 290-292 | 290-293^31^ |
| 5 |  |  | 25 | 96 | 241-243 | 241-243^32^ |
| 6 |  |  | 15 | 95 | 299-301 | 299-302^33^ |
| 7 |  |  | 15 | 95 | 218-220 | 218-221^34^ |
| 8 |  |  | 18 | 94 | 280-282 | 278-281^31^ |
| 9 |  |  | 15 | 89 | 286-288 | 285-287^35^ |
| 10 |  |  | 15 | 94 | 291-293 | 292-294^33^ |

^a^ Reaction conditions: (1) substituted aldehyde (0.4 mmol), (2) dimedone (1 mmol), (3) ammonium acetate (2 mmol), ethanol (4 mL), magnetic nanocatalyst (20 mg), reflux (80 °C). ^b^ Isolated yield.


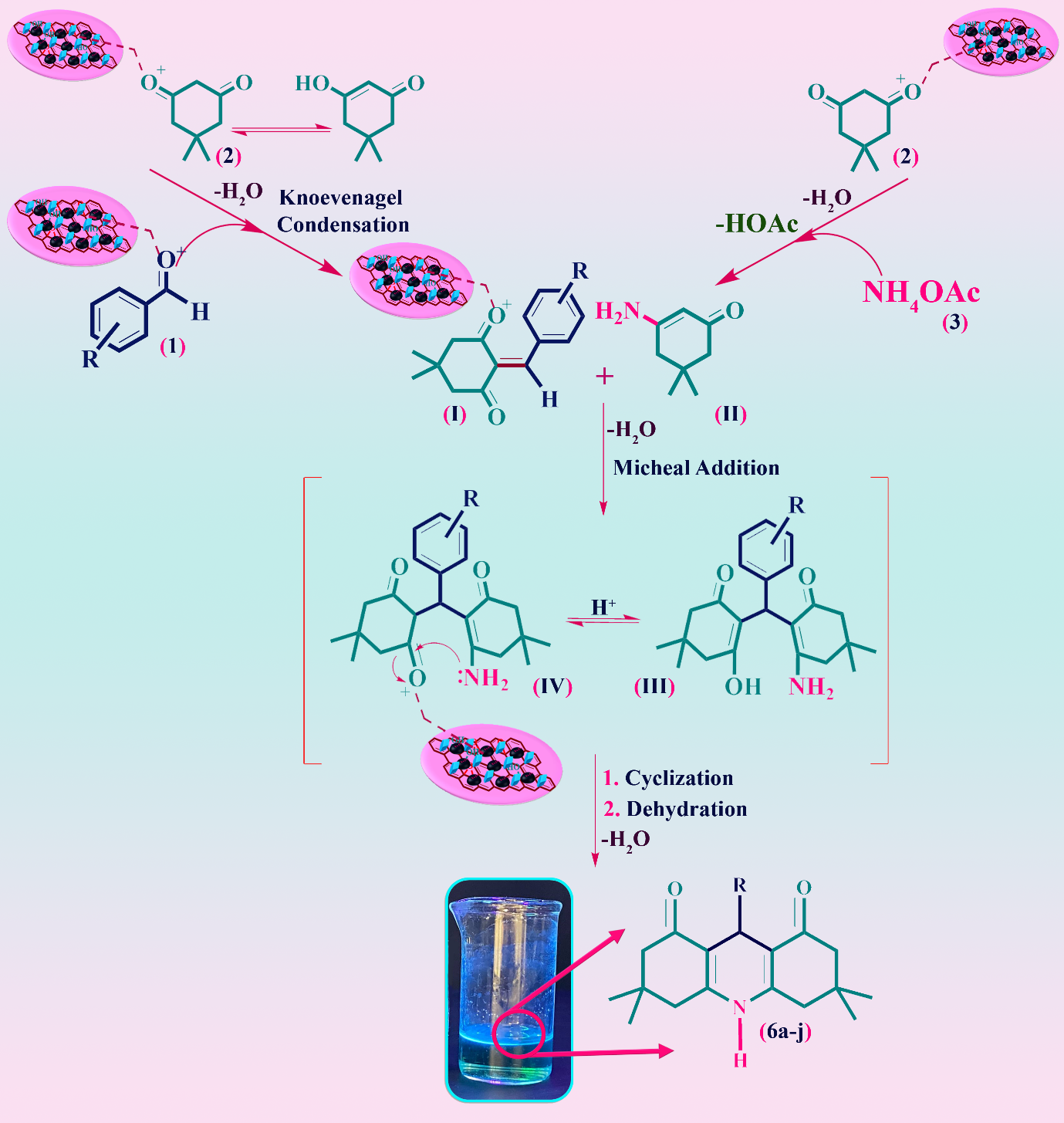


**Fig. S8.** Proposed mechanism and catalytic activity of magnetic rGO aerogel/HKUST-1 nanocomposite in the synthesis of 1,8-dioxo-decahydroacridine derivatives.

| Entry | Catalyst | Catalyst amount | | Solvent | Condition / Temperature (˚C) | Time (h:min) | | Yield^b^ (%) | Ref |
| --- | --- | --- | --- | --- | --- | --- | --- | --- | --- |
| 1 | CeCl_3_.7H_2_O | 0.05 mmol | [bmim][BF_4_] | | 100 °C | | 3:18 | 88 | ^36^ |
| 2 | B(C_6_F_5_)_3_ | 3 mol% | Solvent-free | | R.T. | | 2:48 | 80 | ^37^ |
| 3 | Betainium ionic liquid | 30 mol% | EtOH | | 80 °C | | 3:00 | 90 | ^38^ |
| 4 | CuBr | 5 mol% | CH_3_CN | | 82 °C | | 18:00 | 62 | ^20^ |
| 5 | TMSCl | 20 mmol | CH_3_CN | | Reflux | | 8:00 | 84.1 | ^39^ |
| 6 | Magnetic chitosan-terephthaloyl-creatine nanobiocomposite | 10 mg | EtOH | | 80 °C | | 2:00 | 91 | ^40^ |
| 7 | Salicylic acid | 0.2 eq | Solvent-free | | 80 °C | | 3:00 | 82 | ^21^ |
| 8 | Amberlyst-15 | 200 mg | CH_3_CN | | Reflux | | 5:30 | 82 | ^41^ |
| 9 | Proline | 10 mol% | EtOH | | 65 °C | | 5:00 | 83 | ^42^ |
| 10 | Magnetic rGO aerogel/HKUST-1 nanocomposite | 20 mg | EtOH | | Reflux / (80 °C) | | 15 min | 93 | Present study |

**Table S6.** Comparing the catalytic activity of magnetic rGO aerogel/HKUST-1 nanocomposite with other studies^a^.

^a^ Reaction mixture: benzaldehyde (0.4 mmol), dimedone (1 mmol), ammonium acetate (2 mmol).

^b^ Isolated yield.

**Data characterization of recycled magnetic nanocatalyst**


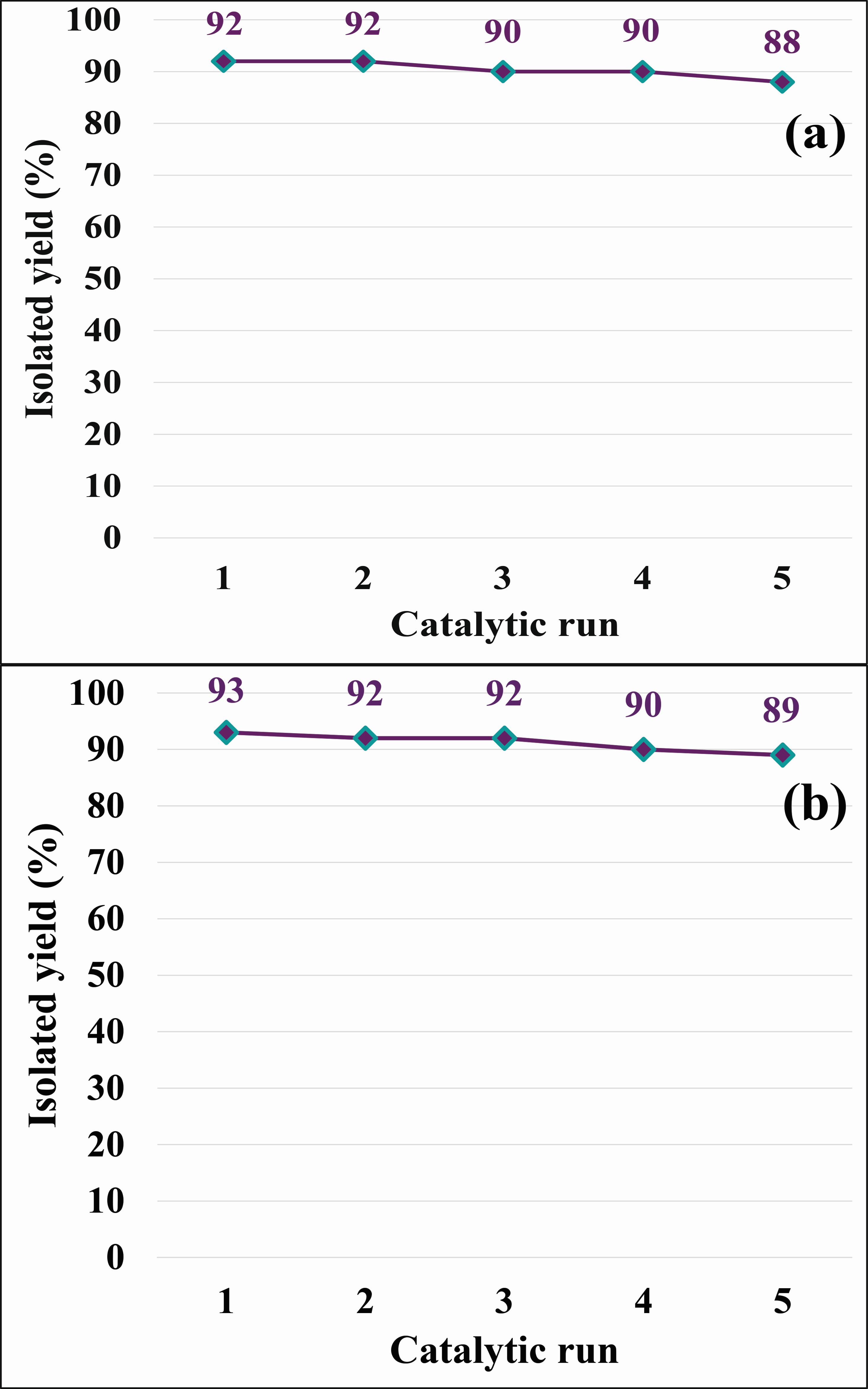


**Fig. S9.** Catalytic recyclability line graph of magnetic rGO aerogel/HKUST-1 nanocomposite (20 mg) in a) the synthesis of product **5a** and b) the synthesis of product **6a**.

**
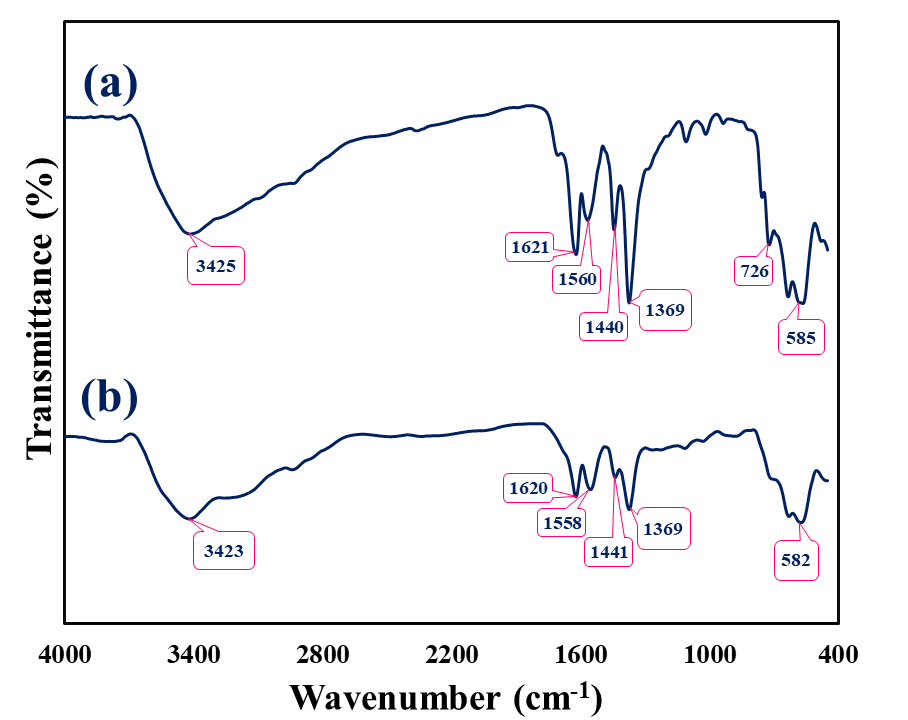
**

**Fig. S10.** FT-IR spectra of (a) magnetic rGO aerogel/HKUST-1 nanocatalyst, (b) recycled magnetic nanocatalyst

**
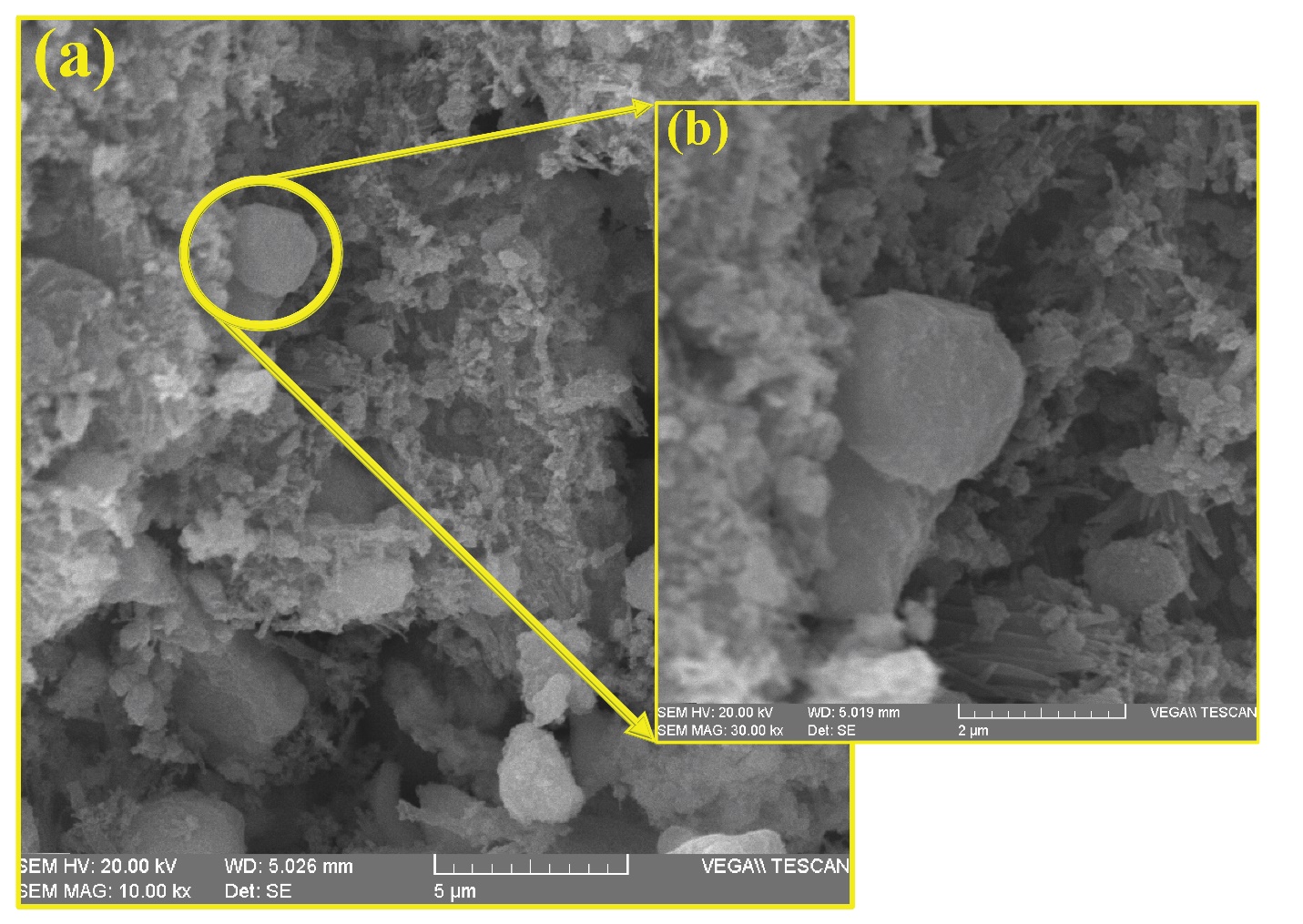
**

**Fig. S11.** SEM images of (a-b) recycled magnetic rGO aerogel/HKUST-1 nanocatalyst

**
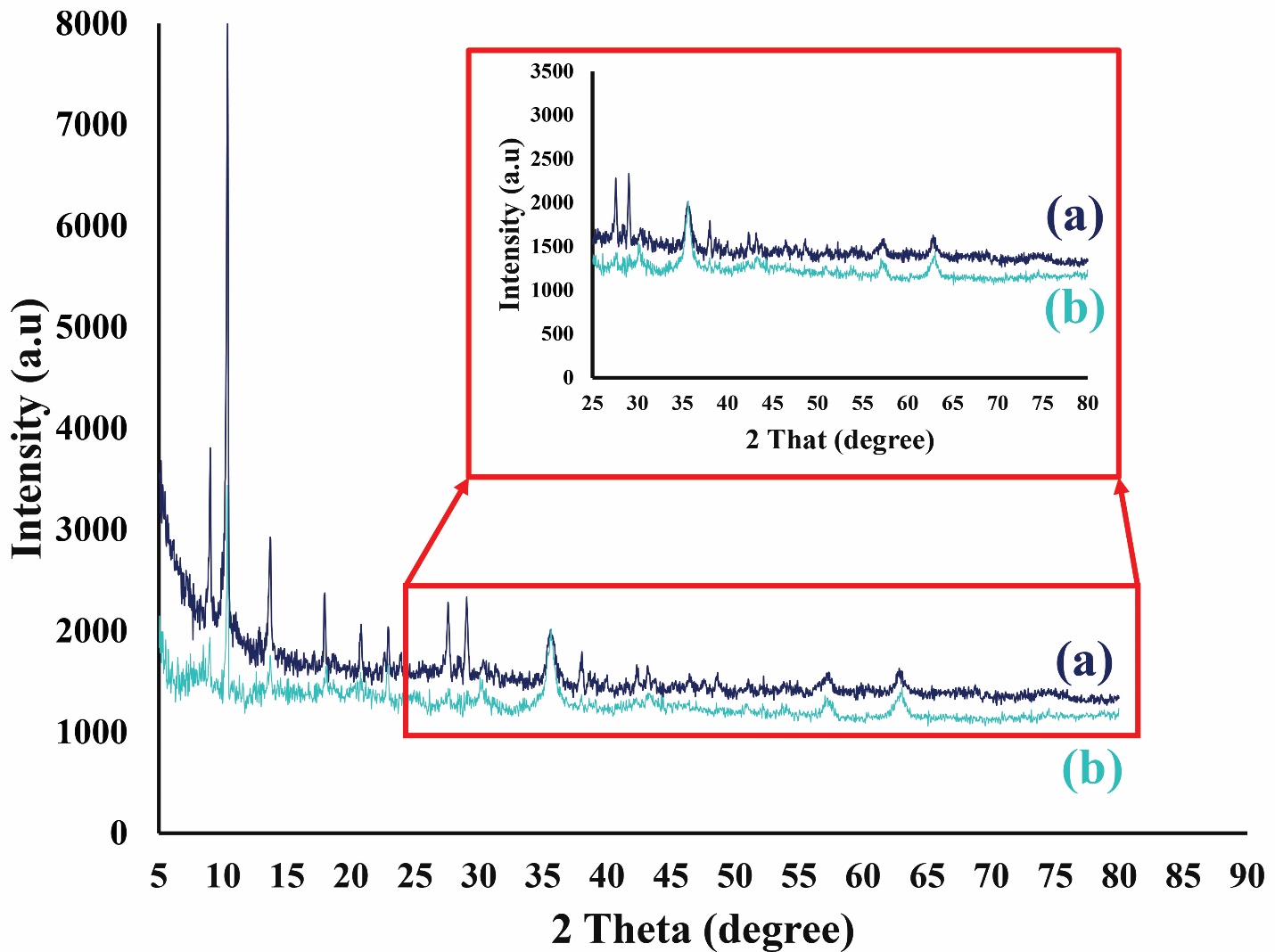
**

**Fig. S12.** XRD patterns of (a) magnetic rGO aerogel/HKUST-1 nanocatalyst, (b) recycled magnetic nanocatalyst

**References**

1. Ghorbani-Choghamarani, A., Heidarnezhad, Z., Tahmasbi, B. & Azadi, G. TEDETA@BNPs as a basic and metal free nanocatalyst for Knoevenagel condensation and Hantzsch reaction. *J. Iran. Chem. Soc.* **15**, 2281-2293 (2018).

2. Yousuf, H. *et al.* Dihydropyridines as potential α-amylase and α-glucosidase inhibitors: synthesis, in vitro and in silico studies. *Bioorg. Chem.* **96**, 103581 (2020).

3. Dhengale, S. D., Naik, V. M., Kolekar, G. B., Rode, C. V. & Anbhule, P. V. Solvent free, environment benign synthesis of 1,4-dihydropyridines and polyhydroquinolines by using heterogeneous Zn/MCM-41 catalyst. *Res. Chem. Intermed.* **47**, 3263-3287 (2021).

4. Ghorbani-Choghamarani, A. & Tahmasbi, B. The first report on the preparation of boehmite silica sulfuric acid and its applications in some multicomponent organic reactions. *New. J. Chem.* **40**, 1205-1212 (2016).

5. Ahad, A. & Farooqui, M. Hydroquinolines via the Hantzsch reaction promoted by SiO_2_-I. *Org. Prep. Proced. Int.* **48**, 371-376 (2016).

6. Ghattali, S. N., Saidi, K. & Khabazzadeh, H. (NH4)_2.5_H_0. 5_PW_12O40_-catalyzed rapid and efficient one-pot synthesis of dihydropyridines via the Hantzsch reaction under solvent-free conditions. *Res. Chem. Intermed.* **40**, 281-291 (2014).

7. Vahdat, S. M. Cu (II) Schiff base complex as a highly efficient catalyst for the synthesis of polyhydroquinoline derivatives via Hantzsch condensation in water. *Comb. Chem. High. Throughput. Screen.* **16**, 782-787 (2013).

8. Khazaei, A., Mahmoudiani Gilan, M. & Sarmasti, N. Magnetic‐based picolinaldehyde–melamine copper complex for the one‐pot synthesis of hexahydroquinolines via Hantzsch four‐component reactions. *Appl. Organomet. Chem.* **32**, e4151 (2018).

9. Mane, P., Shinde, B., Mundada, P., Karale, B. & Burungale, A. Biogenic synthesis of ZnO nanoparticles from Parthenium histerophorus extract and its catalytic activity for building bioactive polyhydroquinolines. *Res. Chem. Intermed.* **47**, 1743-1758 (2021).

10. Otokesh, S. *et al.* A solvent-free synthesis of polyhydroquinolines via Hantzsch multicomponent condensation catalyzed by nanomagnetic-supported sulfonic acid. *S. Afr. J. Chem.* **68**, 15-20 (2015).

11. Yarhosseini, M., Javanshir, S., Dekamin, M. G. & Farhadnia, M. Tetraethylammonium 2-(carbamoyl) benzoate as a bifunctional organocatalyst for one-pot synthesis of Hantzsch 1, 4-dihydropyridine and polyhydroquinoline derivatives. *Monatsh. Chem.* **147**, 1779-1787 (2016).

12. Igder, S., Kiasat, A. R. & Shushizadeh, M. R. Melamine supported on hydroxyapatite-encapsulated-γ-Fe_2_O_3_: a novel superparamagnetic recyclable basic nanocatalyst for the synthesis of 1, 4-dihydropyridines and polyhydroquinolines. *Res. Chem. Intermed.* **41**, 7227-7244 (2015).

13. Vahdat, S. M. *et al.* Synthesis of polyhydroquinoline derivatives via a four-component Hantzsch condensation catalyzed by tin dioxide nanoparticles. *Chinese. J. Catal.* **34**, 758-763 (2013).

14. Rathod, V. N., Bansode, N. D., Thombre, P. B. & Lande, M. K. Efficient one‐pot synthesis of polyhydroquinoline derivatives through the Hantzsch condensation using IRMOF‐3 as heterogeneous and reusable catalyst. *J. Chinese. Chem. Soc.* **68**, 601-609 (2021).

15. Zhang, Q., Ma, X. M., Wei, H. X., Zhao, X. & Luo, J. Covalently anchored tertiary amine functionalized ionic liquid on silica coated nano-Fe_3_O_4_ as a novel, efficient and magnetically recoverable catalyst for the unsymmetrical Hantzsch reaction and Knoevenagel condensation. *RSC. Adv.* **7**, 53861-53870 (2017).

16. Hong, M., Cai, C. & Yi, W.B. Hafnium (IV) bis(perfluorooctanesulfonyl) imide complex catalyzed synthesis of polyhydroquinoline derivatives via unsymmetrical Hantzsch reaction in fluorous medium. *J. Fluor. Chem.* **131**, 111-114 (2010).

17. Choudhury, P., Ghosh, P. & Basu, B. Amine-functionalized graphene oxide nanosheets (AFGONs): an efficient bifunctional catalyst for selective formation of 1,4-dihydropyridines, acridinediones and polyhydroquinolines. *Mol. Divers.* **24**, 283-294 (2020).

18. Wang, L. M. *et al.* Facile Yb(OTf)_3_ promoted one-pot synthesis of polyhydroquinoline derivatives through Hantzsch reaction. *Tetrahedron*. **61**, 1539-1543 (2005).

19. Karade, N. N., Budhewar, V. H., Shinde, S. V. & Jadhav, W. N. L-proline as an efficient organo-catalyst for the synthesis of polyhydroquinoline via multicomponent Hantzsch reaction. *Lett. Org. Chem.* **4**, 16-19 (2007).

20. Sarkar, R., Mukhopadhyay, C. Cross-dehydrogenative regioselective Csp^3^–Csp^2^ coupling of enamino-ketones followed by rearrangement: an amazing formation route to acridine-1,8-dione derivatives. *Org. Biomol. Chem.* **14**, 2706-2715 (2016).

21. Khodja, I. A. *et al.* Solvent-Free Synthesis of dihydropyridines and acridinediones via a salicylic acid–catalyzed Hantzsch multicomponent reaction. *Synth. Commun.* **44**, 959-967 (2014).

22. Xia, J. J. & Zhang, K. H. Synthesis of N-substituted acridinediones and polyhydroquinoline derivatives in refluxing water. *Molecules*. **17**, 5339-5345 (2012).

23. Kumar, A. & Maurya, R. A. Bakers’ yeast catalyzed synthesis of polyhydroquinoline derivatives via an unsymmetrical Hantzsch reaction. *Tetrahedron. Lett.* **48**, 3887-3890 (2007).

24. Saha, M. & Pal, A. K. Palladium(0) nanoparticles: an efficient catalyst for the one-pot synthesis of polyhydroquinolines. *Tetrahedron. lett.* **52**, 4872-4877 (2011).

25. Hajjami, M. & Tahmasbi, B. Synthesis and characterization of glucosulfonic acid supported on Fe_3_O_4_ nanoparticles as a novel and magnetically recoverable nanocatalyst and its application in the synthesis of polyhydroquinoline and 2,3-dihydroquinazolin-4(1 H)-one derivatives. *RSC. Adv.***5**, 59194-59203 (2015).

26. Tamoradi, T., Mousavi, S. M. & Mohammadi, M. Praseodymium(iii) anchored on CoFe_2_O_4_ MNPs: an efficient heterogeneous magnetic nanocatalyst for one-pot, multi-component domino synthesis of polyhydroquinoline and 2,3-dihydroquinazolin-4(1H)-one derivatives. *New. J. Chem.* **44**, 3012-3020 (2020).

27. Ko, S. & Yao, C. F. Ceric ammonium nitrate (CAN) catalyzes the one-pot synthesis of polyhydroquinoline via the Hantzsch reaction. *Tetrahedron*. **62**, 7293-7299 (2006).

28. Srividya, N., Ramamurthy, P., Shanmugasundaram, P. & Ramakrishnan, V. Synthesis, characterization, and electrochemistry of some acridine-1, 8-dione dyes. *J.* *Org. Chem.* **61**, 5083-5089 (1996).

29. Navarro, C. A., Sierra, C. A. & Ochoa-Puentes, C. Evaluation of sodium acetate trihydrate–urea DES as a benign reaction media for the Biginelli reaction. unexpected synthesis of methylenebis (3-hydroxy-5,5-dimethylcyclohex-2-enones), hexahydroxanthene-1,8-diones and hexahydroacridine-1, 8-diones. *RSC. Adv.* **6**, 65355-65365 (2016).

30. Kiani, M. & Mohammadipour, M. Fe_3_O_4_@SiO_2_–MoO_3_H nanoparticles: a magnetically recyclable nanocatalyst system for the synthesis of 1,8-dioxo-decahydroacridine derivatives. *RSC. Adv.* **7**, 997-1007 (2017).

31. Chavan, P. N., Pansare, D. N. & Shelke, R. N. Eco‐friendly, ultrasound‐assisted, and facile synthesis of one‐pot multicomponent reaction of acridine‐1,8(2H, 5H)‐diones in an aqueous solvent. *J. Chinese. Chem. Soc.* **66**, 822-828 (2019).

32. Sehout, I., Boulcina, R., Boumoud, B., Boumoud, T. & Debache, A. Solvent-free synthesis of polyhydroquinoline and 1,8-dioxodecahydroacridine derivatives through the Hantzsch reaction catalyzed by a natural organic acid: a green method. *Synth. Commun.* **47**, 1185-1191 (2017).

33. Amoozadeh, A., Rahmani, S., Bitaraf, M., Abadi, F. B. & Tabrizian, E. Nano-zirconia as an excellent nano support for immobilization of sulfonic acid: a new, efficient and highly recyclable heterogeneous solid acid nanocatalyst for multicomponent reactions. *New. J. Chem.* **40**, 770-780 (2016).

34. Nakhaei, A., Davoodnia, A. & Morsali, A. Extraordinary catalytic activity of a Keplerate-type giant nanoporous isopolyoxomolybdate in the synthesis of 1,8-dioxo-octahydroxanthenes and 1, 8-dioxodecahydroacridines. *Res. Chem. Intermed.* **41**, 7815-7826 (2015).

35. Faisal, M. *et al.* DABCO–PEG ionic liquid-based synthesis of acridine analogous and its inhibitory activity on alkaline phosphatase. *Synth. Commun.* **48**, 462-472 (2018).

36. Fan, X., Li, Y., Zhang, X., Qu, G. & Wang, J. An efficient and green preparation of 9‐arylacridine‐1, 8‐dione derivatives. *Heteroat. Chem.* **18**, 786-790 (2007).

37. Chandrasekhar, S., Rao, Y. S., Sreelakshmi, L., Mahipal, B. & Reddy, C. R. Tris (pentafluorophenyl) borane-catalyzed three-component reaction for the synthesis of 1,8-dioxodecahydroacridines under solvent-free conditions. *Synthesis*. **2008**, 1737-1740 (2008).

38. Zhu, A., Liu, R., Du, C. & Li, L. Betainium-based ionic liquids catalyzed multicomponent Hantzsch reactions for the efficient synthesis of acridinediones. *RSC. Adv.* **7**, 6679-6684 (2017).

39. Kantevari, S., Bantu, R. & Nagarapu, L. TMSCl mediated highly efficient one-pot synthesis of octahydroquinazolinone and 1,8-dioxo-octahydroxanthene derivatives. *Arkivoc*. **16**, 136-148 (2006).

40. Asgharnasl, S., Eivazzadeh-Keihan, R., Radinekiyan, F. & Maleki, A. Preparation of a novel magnetic bionanocomposite based on factionalized chitosan by creatine and its application in the synthesis of polyhydroquinoline, 1,4-dyhdropyridine and 1,8-dioxo-decahydroacridine derivatives. *Int. J. Biol. Macromol.* **144**, 29-46 (2020).

41. Das, B., Thirupathi, P., Mahender, I., Reddy, V. S. & Rao, Y. K. Amberlyst-15: An efficient reusable heterogeneous catalyst for the synthesis of 1, 8-dioxo-octahydroxanthenes and 1, 8-dioxo-decahydroacridines. *J. Mol. Catal. A. Chem.* **247**, 233-239 (2006).

42. Venkatesan, K., Pujari, S. S. & Srinivasan, K. V. Proline-catalyzed simple and efficient synthesis of 1, 8-dioxo-decahydroacridines in aqueous ethanol medium. *Synth. Commun.* **9**, 228-241 (2008).
